# The Effect of Cardiac Resynchronization Therapy on Right Ventricular Function: A Systematic Review and Meta-Analysis

**DOI:** 10.3390/jcm13144173

**Published:** 2024-07-16

**Authors:** Georgios Sidiropoulos, Paschalis Karakasis, Antonios Antoniadis, Athanasios Saplaouras, Theodoros Karamitsos, Nikolaos Fragakis

**Affiliations:** 1 Department of Cardiology, Georgios Papanikolaou General Hospital, Leoforos Papanikolaou, PK 57010 Thessaloniki, Greece; sidiropoulos.georges@gmail.com; 2 2nd Cardiology Department, Hippokration General Hospital, Aristotle University Medical School, Κonstantinoupoleos 49, PK 54642 Thessaloniki, Greece; pakar15@hotmail.com; 3 3rd Cardiology Department, Hippokration General Hospital, Aristotle University Medical School, Κonstantinoupoleos 49, PK 54642 Thessaloniki, Greece; aantoniadis@gmail.com; 4 Onassis Cardiac Surgery Center, Electrophysiology Department, Leoforos Andrea Syggrou 356, PK 17674 Athens, Greece; saplaouras@hotmail.com; 5 1st Cardiology Department, AHEPA University Hospital, Aristotle University Medical School, Kiriakidi 1, PK 54636 Thessaloniki, Greece; tkaramitsos@auth.gr

**Keywords:** cardiac resynchronization therapy response, right ventricular function, tricuspid annular plane systolic excursion, right ventricular global longitudinal strain, right ventricular fractional area change

## Abstract

**Background**: Right ventricular (RV) failure is an important predicting factor regarding overall and event-free survival regardless of baseline left ventricular (LV) function in patients with severe heart failure (HF). Previous studies have indicated that cardiac resynchronization therapy (CRT) improves LV and RV reverse remodeling in patients with systolic dyssynchrony within the left ventricle. However, there is conflicting evidence regarding the role of CRT in RV function. The aim of this systematic review and meta-analysis was to examine the implications of CRT on RV function indices. **Methods**: A systematic literature search was conducted using the MedLine and EMBASE databases and the Cochrane Library from their inception until 18 March 2024. Eligible were studies providing information on RV function indices, both at baseline and after CRT. Evidence was summarized using random-effects meta-analytic models. **Results**: In total, 30 studies were deemed eligible. CRT resulted in a significant improvement in right ventricular fractional area change (mean difference (MD) 5.11%, 95% confidence interval (CI) 2.83 to 7.39), tricuspid annular plane systolic excursion (TAPSE, MD 1.63 mm, 95% CI 1.10 to 2.16), and myocardial systolic excursion velocity (MD 1.85 cm/s, 95% CI 1.24 to 2.47) as well as a significant decrease in pulmonary artery systolic pressure (MD −6.24 mmHg, 95% CI −8.32 to −4.16). A non-significant effect was observed on TAPSE to PASP ratio and right ventricular global longitudinal strain. **Conclusions**: Our meta-analysis demonstrates that CRT is associated with a significant improvement in echocardiographic parameters of RV function. Further investigation is necessary to elucidate how these changes, both independently and in conjunction with LV improvement, impact patients’ long-term prognosis, and to identify the specific patient populations expected to derive the greatest benefit.

## 1. Introduction

Current guidelines strongly recommend cardiac resynchronization therapy (CRT) due to its demonstrated effect in reducing mortality and hospitalizations among specific heart failure (HF) patient cohorts [1]. While the primary goal of CRT is to improve left ventricular function and reduce heart failure symptoms, the impact of CRT on right ventricular (RV) function is also an important consideration. Early studies proposed RV function as an important parameter that foresees the overall and event-free survival of severe HF patients [2,3]. However, there is conflicting evidence regarding the role of CRT in RV function, with some studies suggesting that CRT does not have a clinically significant impact on RV function and dimensions [4,5,6]. Other studies have reported that CRT contributes to RV function improvement and LV and RV reverse remodeling in patients with systolic dyssynchrony within the left ventricle at follow-up [7,8,9,10,11,12]. Sharma established a notable improvement in RV size and function in HF patients which was dependent on age, QRS duration, and baseline left ventricular ejection fraction (LVEF) [13]. In the present meta-analysis, we sought to evaluate the effect of CRT on RV function using additional echocardiographic parameters.

## 2. Methods

This meta-analysis was conducted in accordance with the Preferred Reporting Items for Systematic Review and Meta-Analyses guidelines (PRISMA Statement) [14]. The protocol of this meta-analysis was registered a priori in the Open Science Framework repository (https://osf.io/wb9pj/) and was adhered to without deviations.

### 2.1. Search Strategy

Two independent investigators conducted a comprehensive search in the MEDLINE and Embase databases, as well as the Cochrane Library, from inception to 18 March 2024. No restrictions were imposed (Supplemental Tables S1–S3). The following terms were utilized to gather all pertinent studies: “RV fractional area change”, “RVFAC”, “tricuspid annular plane systolic excursion”, “TAPSE”, “right ventricular function”, “cardiac resynchronization therapy”, and “biventricular pacemaker”. Additionally, the reference lists of identified studies were manually searched.

### 2.2. Eligibility Criteria

We included randomized clinical trials (RCTs) and observational studies that reported RV echocardiographic parameters before and after CRT. Participants were adults (over 18) who met the European Guidelines for CRT at the time of each study. Although all RV parameters were considered, only data on RVFAC (right ventricular fractional area change), RVGLS (right ventricular global longitudinal strain), TAPSE (tricuspid annular plane systolic excursion), S’ (myocardial systolic excursion velocity), PASP (systolic pulmonary artery pressure), and the TAPSE/PASP ratio were sufficient for meta-analysis. We excluded studies written in a language other than English and those involving congenital heart disease cases.

### 2.3. Data Extraction

Subsequent information was retrieved from each study: publication details, patient characteristics (including overall number of patients, mean age, sex percentages, cardiomyopathy category, left ventricular ejection fraction (LVEF), the presence of atrial fibrillation (AF), and the presence of left bundle branch block (LBBB)). Additionally, the type of right RV indices provided in each study, along with the raw data of these indices before and after CRT, were documented (Table 1 and Table 2). For any missing outcome data, the authors of the primary studies were contacted. Data extraction was performed independently by two investigators.

### 2.4. Quality Assessment

The quality assessment of the included studies was conducted using the Newcastle–Ottawa Quality Assessment Scale (NOS) by two independent researchers. The NOS scoring system comprises three sections: participant selection, result comparability, and outcome quality. Each item specified with a number in the Selection and Outcome categories can receive up to one star, while those in the Comparability category can receive up to two stars. The scale ranges from null to nine stars, with studies classified as low quality if the score is less than 5, fair if the score is between 5 and 7, and good if the score is 8 or higher.

### 2.5. Statistical Analysis

Data analysis was conducted with R software v. 4.2 (R Foundation for Statistical Computing, Vienna, Austria). We conducted a quantitative synthesis regarding the difference between RV indices of RVFAC, RVGLS, TAPSE, S’, SPAP, and TAPSE/PASP ratio before and after CRT. Continuous outcome variables were pooled as mean differences (MD) with 95% confidence intervals (CI). We employed a random-effects model with inverse-variance weighting for the analyses. To assess publication bias, we utilized funnel plots and conducted Egger’s test. Subgroup analysis according to the follow-up time and responder/non-responder status was also conducted. The I^2^ index determines the heterogeneity across studies. A P-value of less than 0.05 (two-tailed) was considered statistically significant. Leave-one-out sensitivity analysis sorted by effect size on the association between cardiac resynchronization therapy and change in TAPSE, PASP, TAPSE/PASP, RVFAC, S’, and RVGLS was also performed. For the main analyses with an adequate number of studies, meta-regression analysis was performed to investigate sources of heterogeneity and the effect of possible effect modifiers, namely the year of publication, follow-up time (in years), age (in years), male sex, baseline LVEF, baseline LVEDV, baseline QRS, baseline NYHA class II–III, ischemic cardiomyopathy, diabetes, hypertension, and the use of ACEI/ARBs, beta-blockers, and diuretics on the difference in TAPSE before and after CRT implantation. A similar analysis was performed for RVFAC and PASP. Data regarding other RV function parameters was inadequate for a meta-regression analysis. Statistically significant results required a *p*-value of more than 0.05.

## 3. Results

In total, 30 relevant studies [4,5,7,8,9,10,11,15,16,17,18,19,20,21,22,23,24,25,26,27,28,29,30,31,32,33,34,35,36,37,38,39,40] were included in the analysis (Supplemental Figure S1). The list of excluded studies with rationale is presented in Supplemental Table S4. Of the echocardiographic indices before and after CRT, only RVFAC, RVGLS, TAPSE, S’, SPAP, and TAPSE/PASP had adequate information for quantitative synthesis. Based on NOS, the quality of twenty-nine studies was deemed as fair and one study was deemed as being of good quality (Supplementary Table S5).

**Table 1:** Baseline characteristics of the included studies.

Study	N	Follow-Up (MO)	Imaging Modality		RV Function Parameters					CRT Response Measure	LVEF (%)
				TAPSE	RVGLS	TAPSE/PASP	RVFAC	S’	PASP		
Plata-Corona 2024 [15]	102	6	echo	+	−	−	+	−	+	Improvement of NYHA class ≥ 1 and LVEF ≥ 5% increase	23.7 ± 7.7
Dawood 2023 [16]	63	6	echo 2D and 3D	+	−	+	+	+	+	-	29.07 ± 6.42
Yuecel 2023 [17]	84	12	echo	+	−	−	−	−	−	-	28.37 ± 7.9
Topal 2023 [18]	97	6	echo	+	−	−	−	+	+	LV end-systolic volume reduction ≥ 15% or ≥ 10% increased LVEF	23.8 ± 5.2
Sadeghian 2022 [19]	20	15	echo	+	−	−	−	−	+	-	22.5 ± 5.6
Deaconu 2021 [20]	54	12	echo	+	+	+	+	−	+	LV end-systolic volume reduction > 15%	28.4 ± 1.3
Braganca 2019 [21]	70	6–12	echo	+	−	−	−	+	+	LVEF increase > 5%	26 ± 7
Abdelhamid 2017 [22]	94	6.2 ± 0.4	echo	+	+	−	−	+	+	LV end-systolic volume reduction > 15%	25.9 ± 5.9
Kang 2015 [23,24]	93	6.8 ± 4.6	echo 2D and ED	+	−	−	−	+	+	LV end-systolic volume reduction > 15%	29.88 ± 8.66
Zaborska 2014 [10]	66	6	echo	+	−	−	+	−	−	LV end-systolic volume reduction > 15%	-
Luca 2014 [9]	50	12	echo	+	+	−	+	+	+	LV end-systolic volume reduction > 15%	29.7 ± 7.3
Praus 2012 [27]	58	3	echo	+	−	−	−	−	−	Improvement of QOL, NYHA class (≥ 1 class), and/or 6MWT (>10%) and no hospitalization for HF and no CV death	-
Kusiak 2012 [28]	57	3	echo	+	−	−	+	+	−	LV end-systolic volume reduction > 10%	21.70 ± 4.81
Vitarelli 2011 [29]	81	6	echo	+	−	+	+	−	+	LV end-systolic volume reduction > 15%	-
Aksoy 2011 [11]	54	6	echo	+	−	−	+	−	−	LV end-systolic volume reduction > 10%	24.69 ± 4.01
D’andrea 2009 [30]	110	6	echo	+	−	−	−	−	−	LV end-systolic volume reduction > 15%	-
Scuteri 2009 [7]	44	6	echo	+	−	−	+	−	−	LV end-systolic volume reduction > 15%	23 ± 5
Yuasa 2009 [31]	42	6	echo	−	−	+	−	−	−	LV end-systolic volume reduction > 10%	25.6 ± 6.9
Anter 2010 [32]	48	12	echo	+	−	−	+	−	+	-	24.6 ± 5
Esmaelzadeh 2011 [33]	16	4–7 days	echo	+	−	−	+	−	−	Clinical improvement in NYHA class, 6MWT	18.8 ± 5.5
Knappe 2013 [34]	63	12	echo	−	−	−	+	−	−	LV end-systolic volume reduction > 15%	27 ± 4
Szulik 2011 [8]	90	18	echo	+	−	−	+	−	−	LV end-systolic volume reduction > 15%	24.6 ± 6
Bleeker 2005 [4]	56	6	echo	−	−	−	−	−	+	-	19 ± 6
Cruz 2019 [35]	81	6		+	−	−	−	−	+	LVEF > 10%	26.3 ± 7.1
Donal 2008 [5]	50	3	echo	−	−	−	−	−	+	-	22 ± 6
Martens 2018 [36]	31	6		+	−	−	−	−	−	-	29 ± 5
Sade 2013 [37]	120	32	echo	−	−	−	−	−	+	LV end-systolic volume reduction > 15%	22 ± 6
Park 2016 [38]	300	6	echo	+	+	−	+	−	+	LV end-systolic volume reduction > 15%	24 ± 7
Rapaciuolo 2016 [39]	227	6	echo	+	−	−	+	−	−	LV end-systolic volume reduction ≥ 15% or increased LVEF > 5%	28 ± 6
Leong 2013 [40]	848	6	echo	+	−	−	−	−	−	-	26 ± 8

CRT: cardiac resynchronization therapy, TAPSE: tricuspid annular plane systolic excursion, MO: months, LV: left ventricular, echo: echocardiography, 2D: 2-dimensional, 3D: 3-dimensional, RV: right ventricular, RVFAC: right ventricular fractional area change, PASP: pulmonary artery systolic pressure, LVEF: left ventricular ejection fraction, NYHA: New York Heart Association classification, 6MWT: 6-min walk test, RVGLS: right ventricular global longitudinal strain Tei index: myocardial performance index, S’: myocardial systolic excursion velocity, and QOL: quality of life.

**Table 2:** Baseline characteristics of the included participants.

Study	Mean Age (yr.)	Male (%)	NYHA III/IV (%)	ICM/NICM (%)	LBBB/RBBB (%)	Mean QRS (ms)	DM	HTN	Smoking	b-Blocker	ACEI	Spironolactone/Loop Diuretic
Plata-Corona 2024 [15]	54 ± 18.7	59.8	42.28	36.3/63.7	85.3/14.7	160 ± 29.4	26.5	49	−	−	−	−
Dawood 2023 [16]	62.8 ± 7.23	63.5	74.6	14.3/85.7	−	161.4 ± 25.4	39.7	31.7	6.3	−	−	−
Yuecel 2023 [17]	73 ± 7.9	83	−	62/38	50/8	161.2 ± 22	37.5	75	36	94	78.6	85.2
Topal 2023 [18]	62.8 ± 10.6	75	100	69.1/30.9	−	−	38.1	79.4	26.8		−	−
Sadeghian 2022 [19]	58.5 ± 9.8	80	100	60/40	60/20	160.5 ± 24.4	40	40	25	85	90	75
Deaconu 2021 [20]	64 ± 13.8	58	73.68	28.8/71.2	70.7/29.3	170.27 ± 21.4	43.7	71.8	39	95	−	−
Braganca 2019 [21]	69 ± 9	69	75.7	52.2/47.8	68.1/31.9	162.9 ± 27.4	41.4	80	7.1	84.3	82.9	90
Abdelhamid 2017 [22]	53.7 ± 14.6	79	100	37.3/62.7	−	149.1 ± 10.8	−	−	−	−	−	−
Kang 2015 [23,24]	60.8 ± 12.2	70.97	−	17.2/82.8	45.16/54.84	167.25 ± 30.94	16.13	35.48	−	89.25	86.02	79.57
Zaborska 2014 [10]	66.7 ± 8.9	80	100	75/25	−	−	−	−	−	−	−	−
Luca 2014 [9]	64 ± 6	82	−	78/22	−	−	−	−	−	−	−	−
Praus 2012 [27]	67 ± 9	−	72	57/43	65/35	193 ± 33	−	−	−	78	88	88
Kusiak 2012 [28]	66.4 ± 8.7	95	−	71.9/28.1	66.6/33.4	184.23 ± 28.31	40.4	63.2	−	−	86	87.8
Vitarelli 2011 [29]	−	64	−	60.5/39.5	−	−	−	−	−	75.3	91.3	85.1
Aksoy 2011 [11]	61.9 ± 10.5	79.6	−	70.4/29.6	−	146.64 ± 25.94	35.2	74.1	−	88.8	100	100
D’andrea 2009 [30]	55.4 ± 11.2	53.6	100	45/55	−	149.2 ± 22.1	70	34.5	39.09	83.6	94.5	56.3
Scuteri 2009 [7]	59 ± 10	81	77	31/69	−	157 ± 25	−	−	−	83	95	100
Yuasa 2009 [31]	−	76	100	53/47	52.3/47.7	−	−	−	−	88.09	88.09	−
Anter 2010 [32]	66 ± 12	79.1	−	−	−	−	−	−	−	−	−	−
Esmaeilzadeh 2011 [33]	61.6 ± 10.2	58	−	48.3/56.3	−	143.1 ± 19.5	−	−	−	−	−	−
Knappe 2013 [34]	64 ± 12	81	0	51/49	78/22	162 ± 26	−	45.6	−	84	83	84
Szulik 2011 [8]	57 ± 9	62	100	41.1/58.9	88/12	176 ± 29	22.2	−	−	100	87.8	88.9
Bleeker 2005 [4]	64 ± 11	79	100	52/48	−	176 ± 30	−	−	−	50	93	82
Cruz 2019 [35]	68.10 ± 12.94	67.5	72.5	28.75/67.5	−	−	−	86.2	16.9	87.7	90.9	93.8
Donal 2008 [5]	67 ± 10	75	100	45/55	−	163 ± 28	43.1	−	−	−	−	−
Martens 2018 [36]	66 ± 13	74	29	40/60	83/17	149 ± 28	23	71	−	87	90	71
Sade 2013 [37]	62 ± 13	81	80	59/41	−	−	−	−	−	−	−	−
Park 2016 [38]	66 ± 11	66	88.9	52.7/47.3	64.4/35.6	158 ± 31	−	−	−	84.7	82.3	79.9
Leong 2013 [40]	69 ± 5	78	77	60/40	−	155 ± 33	21	−	−	71	89	83
Rapacciuolo 2016 [39]	70 ± 9	71	54	41/59	81/19	162 ± 26	33	63	−	77	74	83

NYHA: New York Heart Association classification, ICM: ischemic cardiomyopathy, NICM: non-ischemic cardiomyopathy, LBBB: left bundle branch block, RBBB: right bundle branch block, DM: diabetes mellitus, HTN: hypertension, ACEI: ACE inhibitor.

### 3.1. PASP

**Figure 1:**
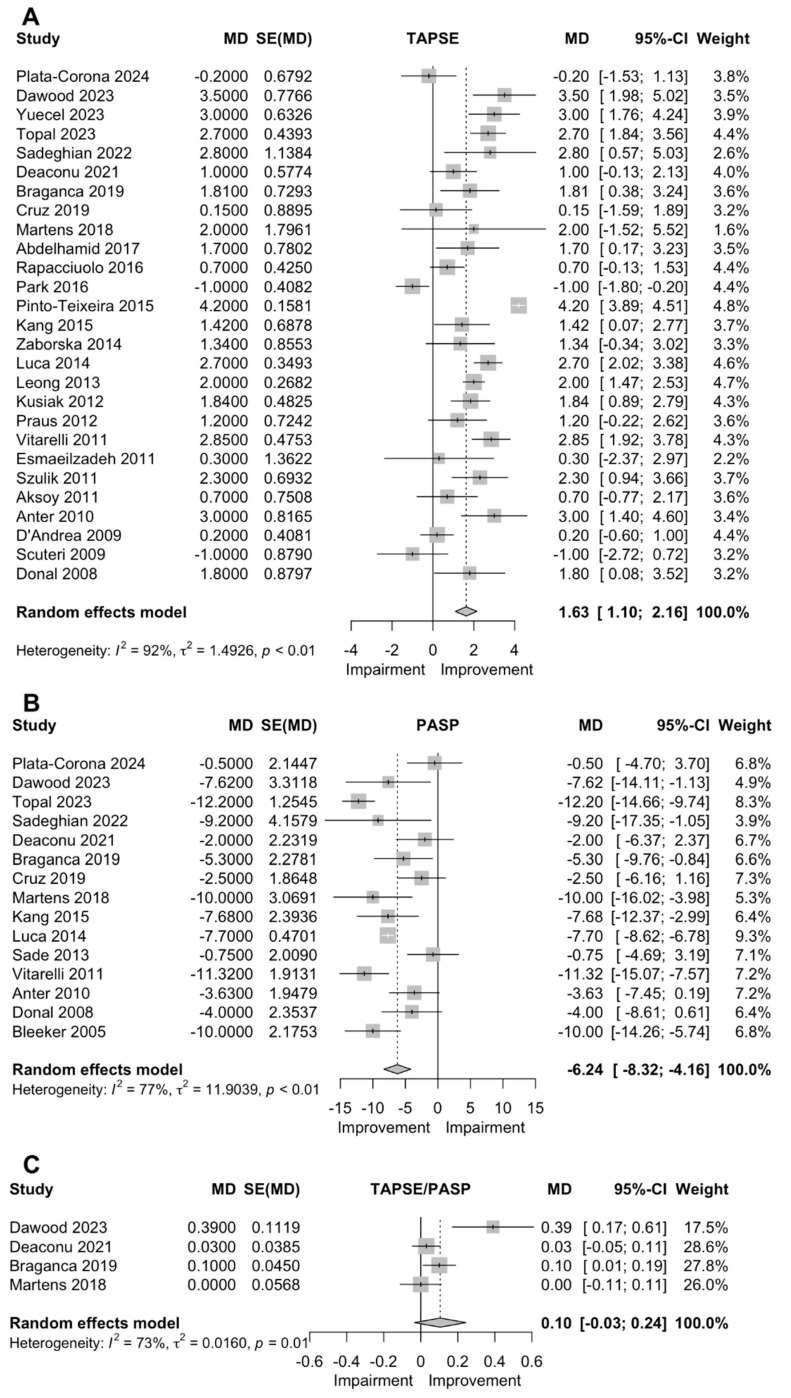
Forest plots of the pooled mean difference in tricuspid annular plane systolic excursion (TAPSE, (**A**)), pulmonary artery systolic pressure (PASP, (**B**)), and TAPSE/PASP ratio (**C**) after cardiac resynchronization therapy [4,5,7,8,9,10,11,15,16,17,18,19,20,21,22,23,24,25,26,27,28,29,30,31,32,33,34,35,36,37,38,39,40]. Abbreviations: CI, confidence interval; MD, mean difference; SE, standard error.

For PASP, 15 studies (n = 1016 patients) were included, and the quantitative synthesis showed a significant decrease in PASP following CRT implantation (MD: −6.24 mmHg (−8.32, −4.16)) (Figure 1).

### 3.2. TAPSE

TAPSE was reported in 27 studies (n = 2914), and a significant increase was observed following CRT implantation (MD: 1.63 mm (1.10, 2.16)) (Figure 1).

### 3.3. TAPSE/PASP

TAPSE/PASP was reported in four studies (n = 242 patients), but there was a non-significant increase (MD: 0.10 (−0.03, 0.24)) (Figure 1).

### 3.4. RVFAC

**Figure 2:**
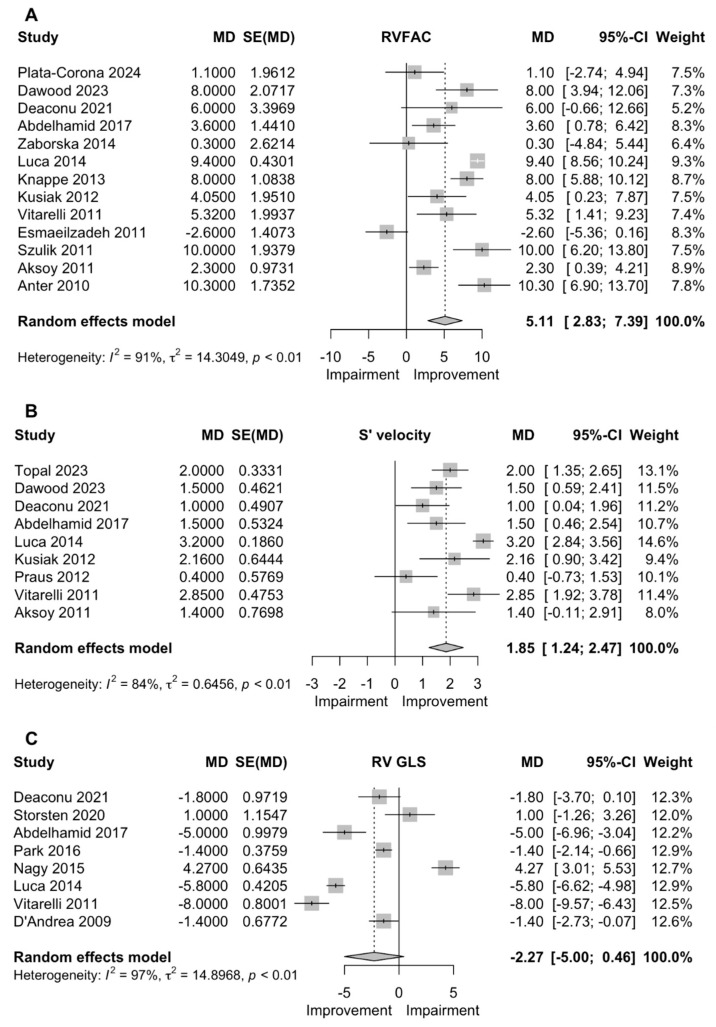
Forest plots of the pooled mean difference in right ventricular fractional shortening (RVFAC, (**A**)), tricuspid annular systolic velocity (S’ velocity, (**B**)) and right ventricular longitudinal strain (RV GLS, (**C**)) after cardiac resynchronization therapy [4,5,7,8,9,10,11,15,16,17,18,19,20,21,22,23,24,25,26,27,28,29,30,31,32,33,34,35,36,37,38,39,40]. Abbreviations: CI, confidence interval; MD, mean difference; SE, standard error.

RVFAC was reported in 13 studies (n = 838 patients) and a significant increase was observed following CRT implantation (MD 5.11% (2.83–7.39)) (Figure 2).

### 3.5. S’

Nine studies (n = 415 patients) were included for the analysis of S’, which revealed a significant increase after CRT (MD: 1.85 cm/s (1.24–2.47)) (Figure 2).

### 3.6. RVGLS

RVGLS was reported in 8 studies (n = 696 patients), but there was a non-significant decrease (MD: −2.27 (−5.00, 0.46)) (Figure 2).

### 3.7. Small Study Effects and Leave-One-Out Sensitivity Analyses

No evidence of small study effects (including publication bias) was observed following the inspection of funnel plots and the Egger’s tests (Supplemental Figures S13–S16). The leave-one-out sensitivity analyses did not show any outliers or influential studies (Supplemental Figures S17–S22).

### 3.8. Subgroup Analyses

The results of the subgroup analyses based on the response in CRT are presented in Supplemental Figures S2–S7. Briefly, CRT resulted in a significant improvement in PASP from baseline for both responders and non-responders. However, significant improvements in the remaining echocardiographic parameters were observed only for the responders to CRT. Subgroup analyses based on the timing of the echocardiographic study indicated that the increase in TAPSE and RVFAC was more profound at 12 months compared to 6 months post CRT implantation (Supplemental Figures S8–S12).

### 3.9. Meta-Regression Analyses

**Figure 3:**
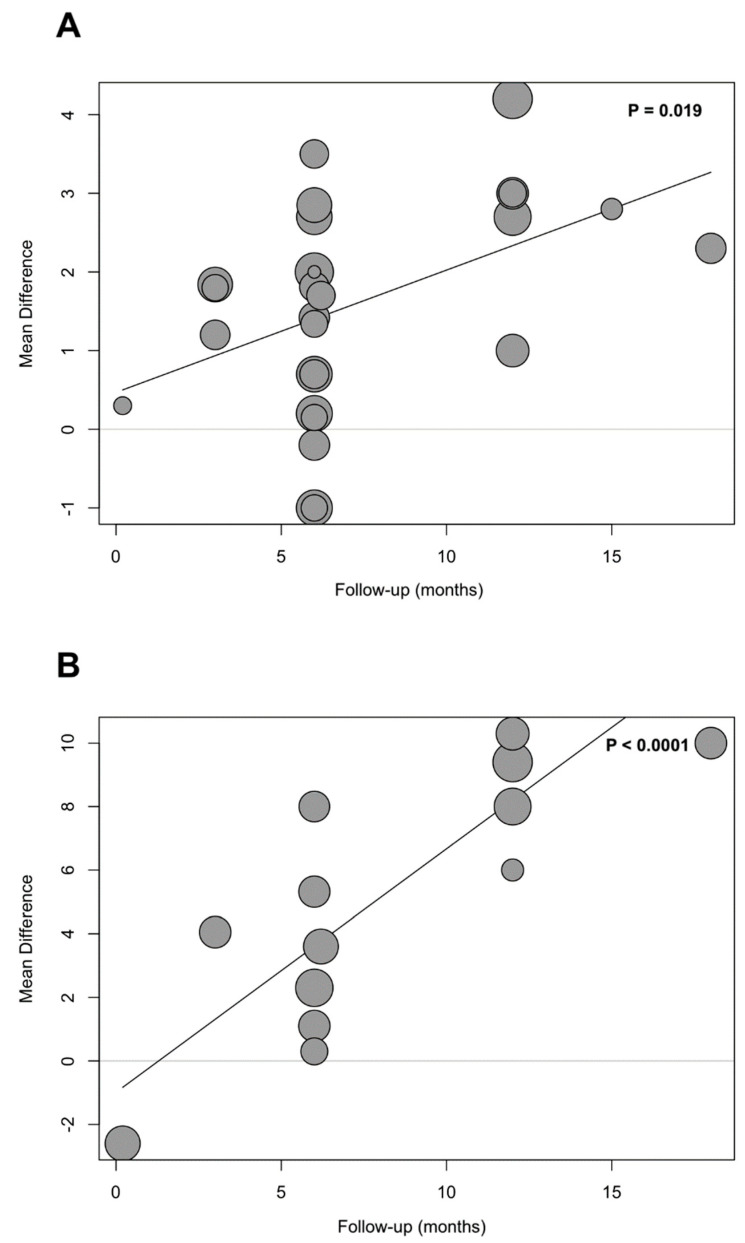
Bubble plots of the meta-regression between TAPSE (**A**) and RVFAC (**B**) and follow-up in the months after CRT implementation.

Most of the performed meta-regression analyses failed to explain a significant proportion of the heterogeneity, as described by R^2^ (Supplemental Tables S6–S8). Studies with longer follow-up periods reported significantly greater improvements in TAPSE and RVFAC (Figure 3). The baseline LV ejection fraction had no significant impact on the post-intervention improvements in the outcomes of interest. Nevertheless, baseline end-systolic and end-diastolic LV volumes were inversely associated with the reduction in PASP following CRT implementation (Supplemental Table S8).

## 4. Discussion

Our metanalysis indicates an improvement in TAPSE, S’, and RVFAC as well as a reduction in SPAP after CRT implantation, demonstrating an improvement in RV function. With regards to RVGLS and TAPSE/PASP, we could not find a significant difference after CRT implantation. We confirmed these findings in a subgroup analysis of studies with a 6- and 12-month follow-up regarding TAPSE increase, studies with a 6- and 12-month follow-up regarding RVFAC increase, studies with a 6- and 12-month follow-up regarding SPAP decrease, and the 6-month follow-up study concerning S’ increase. We could not confirm these findings in a subgroup analysis of studies with a 6- and 12-month follow-up concerning RVGLS increase. The increase in TAPSE and RVFAC was greater at 12 months compared to 6 months. This observed trend toward a continuous improvement of RV function according to follow-up time could be further assessed and potentially confirmed in studies with even longer follow-up periods. In the subgroup analysis according to CRT response status, a significant increase in TAPSE, RVFAC, and S’ was confirmed only in the responders group. A significant decrease in PASP was confirmed both in responders and non-responders. This finding cannot be explained solely by the existing binary terminology of CRT response and underlines the need for the substitution of this term by the concept of disease modification [41]. In this regard, the decrease in PASP could reflect a stabilization of HF or a slowing of LV function deterioration, suggesting partial remission of the disease [41]. The increase in TAPSE after CRT implantation was dependent on follow-up time and male sex, while the increase in RVFAC was dependent on follow-up time, baseline LVEF, QRS width, NYHA class II-III, and the use of ACEI/ARB. PASP decrease was dependent on baseline LVESV, LVEDV, and diuretic use.

The existing data regarding the effect of CRT on RV function are somewhat conflicting. The first evaluation of RV remodeling after CRT was conducted by Bleeker et al. [4]. Significant RV reverse remodeling did not appear immediately after CRT but was observed at follow-up, with a notable decrease in tricuspid valve short axis, long axis, and annulus dimensions, particularly in patients with the largest RV size at baseline. Donal et al. [5] examined the early effects of CRT on RV function using myocardial strain imaging. Although RV dimensions were similar at baseline and the 3-month follow-up, there was an improvement in TAPSE, RV lateral wall basal strain, and mid strain. In a post hoc analysis of the MADIT-CRT trial [6], patients were randomized into two groups: one receiving CRT with a defibrillator (CRT-D) and the other receiving only an implantable cardioverter defibrillator (ICD). The CRT-D group demonstrated significantly greater improvements in RV function and a reduction in tricuspid regurgitation (TR) compared to the ICD-only group at the 1-year follow-up.

In contrast, studies by Scuteri et al. [7] and Boriani et al. [42] did not find any significant changes in RV dimensions and function at 6 and 3 months, respectively, after CRT implantation. Similarly, a substudy of the REVERSE trial by Kjaergaard et al. [43] including patients with NYHA functional class I and II symptoms, found no clinically significant changes in TAPSE and SPAP at 12-month follow-up both in CRT ON and CRT OFF groups, concluding that CRT does not have a clinically significant effect on TAPSE. In addition, an analysis of the CARE-HF trial [44], showed no improvement in RV function (as assessed by changes in TAPSE and RVFAC) and RV structure (as assessed by right ventricular end-systolic area), both in the presence and absence of ischemic heart disease.

In a prospective study of 905 patients by Leong et al. [40], TAPSE increased, and right ventricular systolic pressure (RVSP) decreased at a 6-month follow-up. The improvement of TAPSE was independent of LVEF change and mitral regurgitation (MR) reduction. These findings suggest that the improvement in RV function indices was associated with a decrease in LV filling pressures, which can indirectly affect RV function. On the contrary, LV systolic function amelioration and MR reduction were not associated with RV function improvement. These findings suggest that CRT affects RV function both directly and indirectly through the enhancement of LV diastolic function.

In a single-center study conducted by Abdelhamid et al. [22], 94 patients were divided into volumetric responders (left ventricular end-systolic volume (LVESV) increase by 15% or more) and non-responders with a follow-up period of 6 months. At the end of the follow-up period, only the responders showed a significant reduction in RV transverse and longitudinal dimensions. Additionally, RV systolic function as measured by RVFAC, TAPSE, S’, and RVGLS showed significant improvement only in the responders’ group.

TAPSE is an easily obtainable parameter for evaluating RV longitudinal shortening and correlates well with right ventricular ejection fraction (RVEF) [45]. However, it is important to note that TAPSE has limitations, including its exclusive focus on assessing the longitudinal shortening of the RV free wall without accounting for the contribution of the remaining walls [46]. Additionally, TAPSE is an angle-dependent parameter that is sensitive to changes in preload and afterload. These limitations should be considered when interpreting TAPSE measurements in clinical practice. RVFAC correlates well with RVEF calculated using MRI but relies on clear endocardial border delineation, which can be challenging in an RV with excessive trabeculation and is also influenced by load status [46]. Therefore, while RVFAC can provide valuable insights into right ventricular function, its interpretation should take into consideration the above-mentioned potential limitations.

Due to the complexity of the RV, multiple echocardiographic parameters were used in this study to estimate the effect of CRT on RV function. Systolic excursion velocity, also known as S’, shows good correlations to radionuclide angiography and cardiac magnetic resonance (CMR)-derived RVEF [47]. Right ventricular–arterial coupling (assessed using TAPSE/PASP ratio) has been identified as an independent predictor of mortality in heart failure patients with both reduced and preserved ejection fraction [48]. Furthermore, this ratio demonstrates a strong correlation with exercise capacity, with improvements in RV-arterial coupling (indicated by an increased TAPSE/PASP ratio) observed only as a result of chronic CRT through reverse remodeling during exercise [36].

Lastly, given the emergence of novel cardiac pacing techniques, including conduction system pacing, future research is needed to thoroughly elucidate their effects on right ventricular function indices, independent of improvements in left ventricular function.

## 5. Limitations

This study has certain limitations, primarily attributed to the study design of the published evidence on RV function. First, most of the included studies are observational. There is considerable variability concerning the assessment of particular RV function indices, such as inter-vendor variability in RVGLS analysis. Additionally, some studies had a relatively small number of participants, limiting investigations in specific patient subgroups, and had relatively short follow-up periods. Consequently, future studies with extended follow-up periods are needed to enable a more precise evaluation of the long-term effects of CRT on RV function.

## 6. Conclusions

This meta-analysis shows that CRT implantation improves several echocardiographic parameters of RV function. For some echocardiographic indices, this benefit was linearly associated with the duration of follow-up, suggesting a continuous long-term benefit of CRT on RV function. Of note, even non-responders received a benefit in PASP. Collectively, our data suggest that while the primary goal of CRT is to improve left ventricular function in heart failure patients, there is evidence to suggest that CRT can also have a positive impact on RV function. Ongoing research in this area should aim to identify the patient populations most likely to benefit from CRT and to optimize device implantation and pacing strategies to maximize benefits to RV function.

## Data Availability

The data generated in this research will be shared upon reasonable request directed to the corresponding author.

## Supplementary Materials

The following supporting information can be downloaded at: https://www.mdpi.com/article/10.3390/jcm13144173/s1.

## Abbreviations

AF	atrial fibrillation
CI	confidence intervals
CMR	cardiovascular magnetic resonance imaging
CRT	cardiac resynchronization therapy
CRT-D	cardiac resynchronization therapy defibrillator
HF	heart failure
ICD	implantable cardioverter defibrillator
LV	left ventricle
LBBB	left bundle branch block
LVEF	left ventricular ejection fraction
LVESV	left ventricular end-systolic volume
MD	mean difference
MPI	myocardial performance index (Tei index)
MR	mitral regurgitation
NOS	Newcastle–Ottawa Quality Assessment Score
NYHA	New York Heart Association Functional Classification
PRISMA	Preferred Reporting Items for Systematic Reviews and Meta-Analyses
RCTs	randomized clinical trials
RV	right ventricle
RVEF	right ventricular ejection fraction
RVFAC	right ventricular fractional area change
RVGLS	right ventricular global longitudinal strain
RVOT	right ventricular outflow tract
RVSP	right ventricular systolic pressure
S’	myocardial systolic excursion velocity
SPAP	systolic pulmonary artery pressure
TAPSE	tricuspid annular plane systolic excursion
TR	tricuspid regurgitation
