# Supplementary material for: The Effect of Cardiac Resynchronization Therapy on Right Ventricular Function: A Systematic Review and Meta-Analysis
Source: J Clin Med. 2024 Jul 16;13(14):4173. doi: 10.3390/jcm13144173 (PMC11277751; doi:10.3390/jcm13144173)
Supplement: Supplementary file 1 [file jcm-13-04173-s001.zip › jcm-3084523-supplementary.pdf]

Supplementary Materials

The Effect of Cardiac Resynchronization Therapy on Right Ventricular Function: A Systematic Review and Meta-Analysis

Contents

Supplementary Table S1. Search strategy for MEDLINE (Pubmed) 2

Supplementary Table S2. Search strategy for Cochrane library 3

Supplementary Table S3. Search strategy for Embase 3

Supplementary Figure S1. Preferred Reporting Items for Systematic Reviews and Meta-Analyses (PRISMA) flow diagram. 4

Supplementary Table S4. Table of excluded studies with rationale. 5

References of excluded studies 5

Supplementary Table S5. Quality assessment of included studies (Newcastle-Ottawa scale) 12

Supplementary Figure S2. Subgroup analysis of tricuspid annular plane systolic excursion (TAPSE) based on the response to cardiac resynchronization therapy. 13

Supplementary Figure S3. Subgroup analysis of pulmonary artery systolic pressure (PASP) based on the response to cardiac resynchronization therapy 14

Supplementary Figure S4. Subgroup analysis of TAPSE/PASP ratio based on the response to cardiac resynchronization therapy. 14

Supplementary Figure S5. Subgroup analysis of right ventricular fractional shortening (RVFAC) based on the response to cardiac resynchronization therapy. 15

Supplementary Figure S6. Subgroup analysis of tricuspid annular systolic velocity (S′ velocity) based on the response to cardiac resynchronization therapy 15

Supplementary Figure S7. Subgroup analysis of right ventricular longitudinal strain (RV GLS) based on the response to cardiac resynchronization therapy 16

Supplementary Figure S8. Subgroup analysis of tricuspid annular plane systolic excursion (TAPSE) at 6 and 12 months 16

Supplementary Figure S9. Subgroup analysis of pulmonary artery systolic pressure (PASP) at 6 and 12 months. 17

Supplementary Figure S10. Subgroup analysis of right ventricular fractional shortening (RVFAC) at 6 and 12 months 17

Supplementary Figure S11. Subgroup analysis of tricuspid annular systolic velocity (S′ velocity) at 6 and 12 months 18

Supplementary Figure S12. Subgroup analysis of right ventricular longitudinal strain (RV GLS) at 6 and 12 months. 18

Supplementary Figure S13. Contour- enhanced funnel plot of effect size versus standard error for the association between cardiac resynchronization therapy and change in tricuspid annular plane systolic excursion (TAPSE). 19

Supplementary Figure S14. Contour- enhanced funnel plot of effect size versus standard error for the association between cardiac resynchronization therapy and change in pulmonary artery systolic pressure (PASP). 19

Supplementary Figure S15. Contour- enhanced funnel plot of effect size versus standard error for the association between cardiac resynchronization therapy and change in right ventricular fractional shortening (RVFAC). 20

Supplementary Figure S16. Contour- enhanced funnel plot of effect size versus standard error for the association between cardiac resynchronization therapy and change in tricuspid annular systolic velocity (S′ velocity). 20

Supplementary Figure S17. Leave-one-out sensitivity analysis sorted by effect size on the association cardiac between cardiac resynchronization therapy and change in tricuspid annular plane systolic excursion (TAPSE). 21

Supplementary Figure S18. Leave-one-out sensitivity analysis sorted by effect size on the association cardiac between cardiac resynchronization therapy and change in change in pulmonary artery systolic pressure (PASP). 21

Supplementary Figure S19. Leave-one-out sensitivity analysis sorted by effect size on the association cardiac between cardiac resynchronization therapy and change in change in TAPSE/PASP ratio. 22

Supplementary Figure S20. Leave-one-out sensitivity analysis sorted by effect size on the association cardiac between cardiac resynchronization therapy and change in right ventricular fractional shortening (RVFAC). 22

Supplementary Figure S21. Leave-one-out sensitivity analysis sorted by effect size on the association cardiac between cardiac resynchronization therapy and change in tricuspid annular systolic velocity (S′ velocity). 23

Supplementary Figure S22. Leave-one-out sensitivity analysis sorted by effect size on the association cardiac between cardiac resynchronization therapy and change in right ventricular longitudinal strain (RV GLS). 23

Supplementary Table S6. Meta-regression analysis on tricuspid annular plane systolic excursion (TAPSE) 24

Supplementary Table S7. Meta-regression analysis on right ventricular fractional shortening (RVFAC) 24

Supplementary Table S8. Meta-regression analysis on pulmonary artery systolic pressure (PASP) 25

Supplementary Table S9. PRISMA 2020 Checklist 26

Table S1. Search strategy for MEDLINE (Pubmed).

**((((FAC) OR (fractional area change)) OR ((tricuspid annular plane systolic excursion) OR ((right ventricular function[MeSH Terms]) OR (right ventricular function)))) OR ((right ventricular function[MeSH Terms]) OR (right ventricular function))) AND ((cardiac resynchronization device) OR (((biventricular pacemaker[MeSH Terms]) OR (biventricular pacemaker)) OR (((cardiac resynchronization therapy*[MeSH Terms]) OR (cardiac resynchronization therapy[MeSH Terms])) OR (cardiac resynchronization therapy))))** Sort by: **Publication Date** ("FAC"[All Fields] OR ("fractional"[All Fields] AND ("geographic locations"[MeSH Terms] OR ("geographic"[All Fields] AND "locations"[All Fields]) OR "geographic locations"[All Fields] OR "area"[All Fields]) AND ("change"[All Fields] OR "changed"[All Fields] OR "changes"[All Fields] OR "changing"[All Fields] OR "changings"[All Fields])) OR ((("tricuspid"[All Fields] OR "tricuspidal"[All Fields] OR "tricuspidization"[All Fields]) AND "annular"[All Fields] AND ("aircraft"[MeSH Terms] OR "aircraft"[All Fields] OR "plane"[All Fields] OR "planes"[All Fields]) AND ("systole"[MeSH Terms] OR "systole"[All Fields] OR "systoles"[All Fields] OR "systolic"[All Fields] OR "systolically"[All Fields]) AND ("excursion"[All Fields] OR "excursions"[All Fields])) OR ("ventricular function, right"[MeSH Terms] OR ("ventricular function, right"[MeSH Terms] OR ("ventricular"[All Fields] AND "function"[All Fields] AND "right"[All Fields]) OR "right ventricular function"[All Fields] OR ("right"[All Fields] AND "ventricular"[All Fields] AND "function"[All Fields])))) OR ("ventricular function, right"[MeSH Terms] OR ("ventricular function, right"[MeSH Terms] OR ("ventricular"[All Fields] AND "function"[All Fields] AND "right"[All Fields]) OR "right ventricular function"[All Fields] OR ("right"[All Fields] AND "ventricular"[All Fields] AND "function"[All Fields])))) AND ((("cardiac resynchronization therapy"[MeSH Terms] OR ("cardiac"[All Fields] AND "resynchronization"[All Fields] AND "therapy"[All Fields]) OR "cardiac resynchronization therapy"[All Fields] OR ("cardiac"[All Fields] AND "resynchronization"[All Fields]) OR "cardiac resynchronization"[All Fields]) AND ("device s"[All Fields] OR "equipment and supplies"[MeSH Terms] OR ("equipment"[All Fields] AND "supplies"[All Fields]) OR "equipment and supplies"[All Fields] OR "device"[All Fields] OR "instrumentation"[MeSH Subheading] OR "instrumentation"[All Fields] OR "devices"[All Fields])) OR (("biventricular"[All Fields] AND "pacemaker, artificial"[MeSH Terms]) OR ("biventricular"[All Fields] AND ("pacemaker s"[All Fields] OR "pacemaker, artificial"[MeSH Terms] OR ("pacemaker"[All Fields] AND "artificial"[All Fields]) OR "artificial pacemaker"[All Fields] OR "pacemaker"[All Fields] OR "pacemakers"[All Fields] OR "pacemaking"[All Fields])) OR ("cardiac resynchronization therapy*"[MeSH Terms] OR "cardiac resynchronization therapy"[MeSH Terms] OR ("cardiac resynchronization therapy"[MeSH Terms] OR ("cardiac"[All Fields] AND "resynchronization"[All Fields] AND "therapy"[All Fields]) OR "cardiac resynchronization therapy"[All Fields]))))
Results: 947

Table S2. Search strategy for Cochrane library.

#1	cardiac resynchronization therapy	2188
#2	MeSH descriptor: [Cardiac Resynchronization Therapy] explode all trees	609
#3	#1 or #2	2188
#4	biventricular pacemaker	184
#5	MeSH descriptor: [Cardiac Resynchronization Therapy Devices] explode all trees	148
#6	#4 or #5	322
#7	#3 or #6	2255
#8	right ventricular function	2368
#9	MeSH descriptor: [Ventricular Function, Right] explode all trees	342
#10	tricuspid annular plane systolic excursion	241
#11	TAPSE	271
#12	fractional area change	355
#13	FAC	1186
#14	#8 or #9 or#10 or#11 or #12 or #13	3869
#15	#7 and #14	450

Table S3. Search strategy for Embase.

('**cardiac resynchronization therapy**'/exp OR '**cardiac resynchronization therapy**' OR '**biventricular pacemaker**'/exp OR '**biventricular pacemaker**' OR '**cardiac resynchronization therapy device**'/exp OR '**cardiac resynchronization therapy device**') AND ('**right ventricular function**' OR '**tapse**' OR '**tricuspid annular plane systolic excursion**'/exp OR '**tricuspid annular plane systolic excursion**' OR '**fac**' OR '**fractional area change**'/exp OR '**fractional area change**')
Results: 305

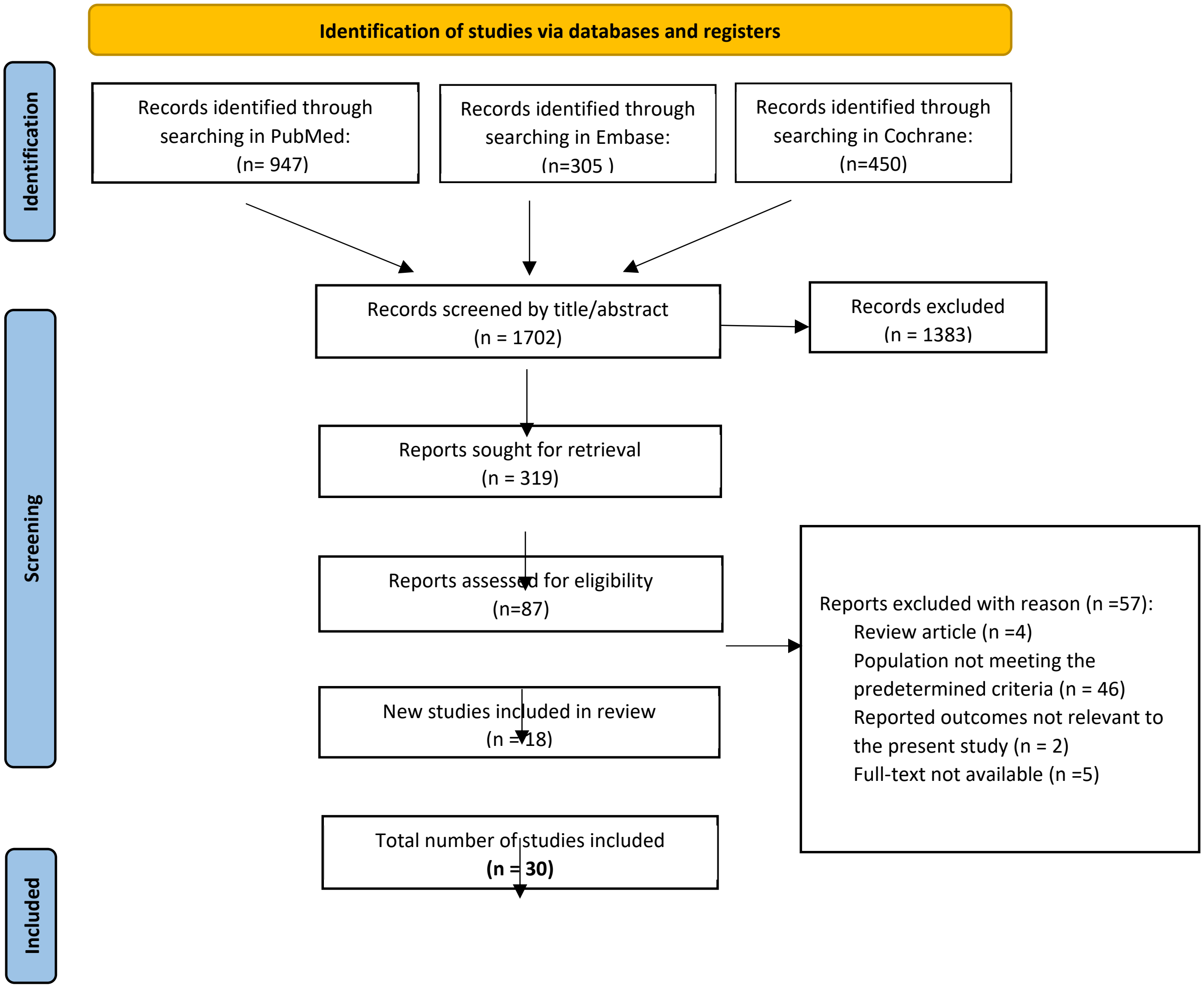

Figure S1. Preferred Reporting Items for Systematic Reviews and Meta-Analyses (PRISMA) flow diagram.

Table S4. Table of excluded studies with rationale.

Reason for exclusion (n=60)	References of excluded studies
Population not meeting the predetermined criteria (n = 46)	[1]-[46]
Reported outcomes not relevant to the present study (n =2)	[47]-[48]
Full-text not available (n =5)	[49]-[53]
Review article (n = 4)	[54]-[57]

References of excluded studies

[1] Valzania C, Biffi M, Bonfiglioli R, Fallani F, Martignani C, Diemberger I, Ziacchi M, Frisoni J, Tomasi L, Fanti S, Rapezzi C, Boriani G. Effects of cardiac resynchronization therapy on right ventricular function during rest and exercise, as assessed by radionuclide angiography, and on NT-proBNP levels. J Nucl Cardiol. 2019 Feb;26(1):123-132. doi: 10.1007/s12350-017-0971-3. Epub 2017 Jun 30. PMID: 28667454.
[2] Schmeisser A, Rauwolf T, Ghanem A, Groscheck T, Adolf D, Grothues F, Fischbach K, Kosiek O, Huth C, Kropf S, Lange S, Luani B, Smid J, Schäfer MH, Schreiber J, Tanev I, Wengler F, Yeritsyan NB, Steendijk P, Braun-Dullaeus RC. Right heart function interacts with left ventricular remodeling after CRT: A pressure volume loop study. Int J Cardiol. 2018 Oct 1;268:156-161. doi: 10.1016/j.ijcard.2018.03.026. Epub 2018 Mar 8. PMID: 29548538.

[3] Petrovic M, Petrovic M, Milasinovic G, Vujisic Tesic B, Trifunovic D, Petrovic O, Nedeljkovic I, Petrovic I, Banovic M, Boricic-Kostic M, Petrovic J, Arena R, Popovic D. Gauging the response to cardiac resynchronization therapy: The important interplay between predictor variables and definition of a favorable outcome. Echocardiography. 2017 Mar;34(3):371-375. doi: 10.1111/echo.13453. Epub 2017 Jan 11. PMID: 28075037.

[4] Helsen F, Van De Bruaene A, Gabriels C, Claeys M, Troost E, Vörös G, Willems R, Voigt JU, Budts W. Prognostic significance of improvement in right ventricular systolic function during cardiac resynchronization therapy. Acta Cardiol. 2017 Jun;72(3):267-275. doi: 10.1080/00015385.2017.1305177. Epub 2017 Mar 31. PMID: 28636525.

[5] van Everdingen WM, Walmsley J, Cramer MJ, van Hagen I, De Boeck BWL, Meine M, Delhaas T, Doevendans PA, Prinzen FW, Lumens J, Leenders GE. Echocardiographic Prediction of Cardiac Resynchronization Therapy Response Requires Analysis of Both Mechanical Dyssynchrony and Right Ventricular Function: A Combined Analysis of Patient Data and Computer Simulations. J Am Soc Echocardiogr. 2017 Oct;30(10):1012-1020.e2. doi: 10.1016/j.echo.2017.06.004. Epub 2017 Aug 8. PMID: 28801203.

[6] Abreu A, Oliveira M, Silva Cunha P, Santa Clara H, Santos V, Portugal G, Rio P, Soares R, Moura Branco L, Alves M, Papoila AL, Ferreira R, Mota Carmo M; BETTER-HF investigators. Predictors of response to cardiac resynchronization therapy: A prospective cohort study. Rev Port Cardiol. 2017 Jun;36(6):417-425. English, Portuguese. doi: 10.1016/j.repc.2016.10.010. Epub 2017 May 27. PMID: 28554585.

[7] Nagy VK, Széplaki G, Apor A, Kutyifa V, Kovács A, Kosztin A, Becker D, Boros AM, Gellér L, Merkely B. Role of Right Ventricular Global Longitudinal Strain in Predicting Early and Long-Term Mortality in Cardiac Resynchronization Therapy Patients. PLoS One. 2015 Dec 23;10(12):e0143907. doi: 10.1371/journal.pone.0143907. PMID: 26700308; PMCID: PMC4689553.

[8] Doyle CL, Huang DT, Moss AJ, Solomon SD, Campbell P, McNitt S, Polonsky S, Barsheshet A, Aktas M, Tompkins C, Zareba W, Goldenberg I. Response of right ventricular size to treatment with cardiac resynchronization therapy and the risk of ventricular tachyarrhythmias in MADIT-CRT. Heart Rhythm. 2013 Oct;10(10):1471-7. doi: 10.1016/j.hrthm.2013.07.029. Epub 2013 Jul 19. PMID: 23872690.

[9] Campbell P, Takeuchi M, Bourgoun M, Shah A, Foster E, Brown MW, Goldenberg I, Huang DT, McNitt S, Hall WJ, Moss A, Pfeffer MA, Solomon SD; Multicenter Automatic Defibrillator Implantation Trial With Cardiac Resynchronization Therapy (MADIT-CRT) Investigators. Right ventricular function, pulmonary pressure estimation, and clinical outcomes in cardiac resynchronization therapy. Circ Heart Fail. 2013 May;6(3):435-42. doi: 10.1161/CIRCHEARTFAILURE.112.000127. Epub 2013 Mar 22. PMID: 23524528.

[10] Damy T, Ghio S, Rigby AS, Hittinger L, Jacobs S, Leyva F, Delgado JF, Daubert JC, Gras D, Tavazzi L, Cleland JG. Interplay between right ventricular function and cardiac resynchronization therapy: an analysis of the CARE-HF trial (Cardiac Resynchronization-Heart Failure). J Am Coll Cardiol. 2013 May 28;61(21):2153-60. doi: 10.1016/j.jacc.2013.02.049. Epub 2013 Mar 26. PMID: 23541971.

[11] Domenichini G, Burri H, Valzania C, Gavaruzzi G, Fallani F, Biffi M, Sunthorn H, Diemberger I, Martignani C, Foulkes H, Fleury E, Boriani G. QRS pattern and improvement in right and left ventricular function after cardiac resynchronization therapy: a radionuclide study. BMC Cardiovasc Disord. 2012 Apr 11;12:27. doi: 10.1186/1471-2261-12-27. PMID: 22494365; PMCID: PMC3352038.

[12] McLeod CJ, Shen WK, Rea RF, Friedman PA, Hayes DL, Wokhlu A, Webster TL, Wiste HJ, Hodge DO, Bradley DJ, Hammill SC, Packer DL, Cha YM. Differential outcome of cardiac resynchronization therapy in ischemic cardiomyopathy and idiopathic dilated cardiomyopathy. Heart Rhythm. 2011 Mar;8(3):377-82. doi: 10.1016/j.hrthm.2010.11.013. Epub 2010 Nov 9. Erratum in: Heart Rhythm. 2011 Apr;8(4):640. PMID: 21070886.

[13] Kjaergaard J, Ghio S, St John Sutton M, Hassager C. Tricuspid annular plane systolic excursion and response to cardiac resynchronization therapy: results from the REVERSE trial. J Card Fail. 2011 Feb;17(2):100-7. doi: 10.1016/j.cardfail.2010.09.002. Epub 2010 Nov 19. PMID: 21300298.

[14] Alpendurada F, Guha K, Sharma R, Ismail TF, Clifford A, Banya W, Mohiaddin RH, Pennell DJ, Cowie MR, McDonagh T, Prasad SK. Right ventricular dysfunction is a predictor of non-response and clinical outcome following cardiac resynchronization therapy. J Cardiovasc Magn Reson. 2011 Oct 31;13(1):68. doi: 10.1186/1532-429X-13-68. PMID: 22040270; PMCID: PMC3217913.

[15] Solomon SD, Foster E, Bourgoun M, Shah A, Viloria E, Brown MW, Hall WJ, Pfeffer MA, Moss AJ; MADIT-CRT Investigators. Effect of cardiac resynchronization therapy on reverse remodeling and relation to outcome: multicenter automatic defibrillator implantation trial: cardiac resynchronization therapy. Circulation. 2010 Sep 7;122(10):985-92. doi: 10.1161/CIRCULATIONAHA.110.955039. Epub 2010 Aug 23. PMID: 20733097.

[16] Wang DM, Han YL, Zang HY, Yu HB, Zhou WW, Zhang DH, Tian Y. [Incidence and causes of nonresponse to cardiac resynchronization therapy in patients with congestive heart failure]. Zhonghua Xin Xue Guan Bing Za Zhi. 2010 Oct;38(10):895-900. Chinese. PMID: 21176632.

[17] Burri H, Domenichini G, Sunthorn H, Fleury E, Stettler C, Foulkes I, Shah D. Right ventricular systolic function and cardiac resynchronization therapy. Europace. 2010 Mar;12(3):389-94. doi: 10.1093/europace/eup401. Epub 2009 Dec 4. PMID: 19966321.

[18] Cappelli F, Cristina Porciani M, Ricceri I, Perrotta L, Ricciardi G, Pieragnoli P, Paladini G, Michelucci A, Padeletti L. Tricuspid annular plane systolic excursion evaluation improves selection of cardiac resynchronization therapy patients. Clin Cardiol. 2010 Sep;33(9):578-82. doi: 10.1002/clc.20806. PMID: 20842743; PMCID: PMC6653600.

[19] Ghio S, Freemantle N, Scelsi L, Serio A, Magrini G, Pasotti M, Shankar A, Cleland JG, Tavazzi L. Long-term left ventricular reverse remodelling with cardiac resynchronization therapy: results from the CARE-HF trial. Eur J Heart Fail. 2009 May;11(5):480-8. doi: 10.1093/eurjhf/hfp034. Epub 2009 Mar 14. PMID: 19287017.

[20] Faran A, Dabrowska-Kugacka A, Lewicka-Nowak E, Tybura S, Zieba B, Daniłowicz-Szymanowicz L, Krzymińska-Stasiuk E, Kempa M, Kogut K, Raczak G. Echocardiographic evaluation of patients with severe heart failure and impairment of intraventricular conduction following cardiac resynchronisation therapy. Kardiol Pol. 2008 Apr;66(4):396-403; discussion 404-5. PMID: 18473268.

[21] Rajagopalan N, Suffoletto MS, Tanabe M, Miske G, Thomas NC, Simon MA, Bazaz R, Gorcsan J 3rd, López-Candales A. Right ventricular function following cardiac resynchronization therapy. Am J Cardiol. 2007 Nov 1;100(9):1434-6. doi: 10.1016/j.amjcard.2007.06.037. Epub 2007 Aug 21. PMID: 17950803.

[22] Field ME, Solomon SD, Lewis EF, Kramer DB, Baughman KL, Stevenson LW, Tedrow UB. Right ventricular dysfunction and adverse outcome in patients with advanced heart failure. J Card Fail. 2006 Oct;12(8):616-20. doi: 10.1016/j.cardfail.2006.06.472. PMID: 17045180.

[23] Boriani G, Fallani F, Martignani C, Biffi M, Saporito D, Greco C, Ziacchi M, Levorato M, Pontone G, Valzania C, Diemberger I, Franchi R, Branzi A. Cardiac resynchronization therapy: effects on left and right ventricular ejection fraction during exercise. Pacing Clin Electrophysiol. 2005 Jan;28 Suppl 1:S11-4. doi: 10.1111/j.1540-8159.2005.00005.x. PMID: 15683474.

[24] Munclinger MJ, Thornton AS, Wasiak MM. Biventricular pacing for heart failure alters electro-mechanical coupling of both ventricles. Cardiovasc J S Afr. 2005 Jul-Aug;16(4):220-6. PMID: 16211127.

[25] Lenom V, Materne P, Hoffer E, Lecoq E, Désiron Q, Waucquez JL, Boland J. Resynchronisation ventriculaire dans la décompensation cardiaque réfractaire [Clinical value of cardiac resynchronization therapy in patients with congestive heart failure]. Rev Med Liege. 2005 Feb;60(2):101-8. French. PMID: 15819373.

[26] Santos JF, Caetano F, Parreira L, Madeira J, Cardoso P, Fonseca N, Segurado F, Soares LN, Inês L. Effects of cardiac resynchronization therapy on right ventricular function--evaluation with tissue Doppler echocardiography. Rev Port Cardiol. 2003 Nov;22(11):1347-55. English, Portuguese. PMID: 14768490.

[27] Galli E, Le Rolle V, Smiseth OA, Duchenne J, Aalen JM, Larsen CK, Sade EA, Hubert A, Anilkumar S, Penicka M, Linde C, Leclercq C, Hernandez A, Voigt JU, Donal E. Importance of Systematic Right Ventricular Assessment in Cardiac Resynchronization Therapy Candidates: A Machine Learning Approach. J Am Soc Echocardiogr. 2021 May;34(5):494-502. doi: 10.1016/j.echo.2020.12.025. Epub 2021 Jan 7. PMID: 33422667.

[28] C Jenei, E Papp, M Clemens, Z Csanadi, P797 Assessment of right ventricular systolic function with 3D echocardiography following cardiac resynchronization therapy, *European Heart Journal - Cardiovascular Imaging*, Volume 21, Issue Supplement_1, January 2020, jez319.453, https://doi.org/10.1093/ehjci/jez319.453

[29] P C Kahr, P Kaufmann, J Kuster, J Tonko, A Breitenstein, A Flammer, F Ruschitzka, J Steffel, S Winnik, P4525
Differential effect of CRT in ischemic and non-ischemic cardiomyopathy: longterm follow-up data from a single center cohort study, *European Heart Journal*, Volume 40, Issue Supplement_1, October 2019, ehz745.0918, https://doi.org/10.1093/eurheartj/ehz745.0918

[30] Loutfi, M., Nawar, M., Eltahan, S., & Elhoda, A. (2016). Predictors of response to cardiac resynchronization therapy in chronic heart failure patients. *The Egyptian Heart Journal*, 68, 227-236. https://doi.org/10.1016/J.EHJ.2016.01.001.

[31] Saeid, Ali & Sardari, Akram & Ejmalian, G. & Lotfi-Tokaldany, Masoumeh & Sahebjam, Mohammad & Safir-Mardanloo, Azam & Yaminisharif, Ahmad & Montazeri, Mahdi & Jalali, A. & Sadeghian, Hakimeh. (2014). Predictors of response to cardiac resynchronization therapy in heart failure patients. Experimental and Clinical Cardiology. 20. 3982-3990.

[32] C. Aggeli, E. Poulidakis, I. Felekos, S. Sideris, P. Dilaveris, K. Gatzoulis, S. Plitaria, C. Stefanadis, Can right ventricular performance status affect responsiveness to cardiac resynchronization therapy?, *European Heart Journal*, Volume 34, Issue suppl_1, 1 August 2013, P5760, https://doi.org/10.1093/eurheartj/eht310.P5760

[33] Petrovic M, Petrovic MT, Milasinovic G, Vujisic-Tesic B, Trifunovic D, Nedeljkovic I, Calovic Z, Ivanovic B, Tesic M, Boricic M, Petrovic O, Petrovic IM, Banovic M, Draganic G, Ostojic M. Prediction of a good response to cardiac resynchronization therapy in patients with severe dilated cardyomyopathy: could conventional echocardiography be the answer after all? Echocardiography. 2012 Mar;29(3):267-75. doi: 10.1111/j.1540-8175.2011.01576.x. Epub 2011 Nov 28. PMID: 22118412.

[34] Cardiac Resynchronisation Therapy: A Randomised Trial of Factory or Echocardiographic Settings for Optimum Response Cobb, Vanessa et al. Heart, Lung and Circulation, Volume 22, Issue 9, 717 – 723

[35] Ahmed I, Loudon BL, Abozguia K, Cameron D, Shivu GN, Phan TT, Maher A, Stegemann B, Chow A, Marshall H, Nightingale P, Leyva F, Vassiliou VS, McKenna WJ, Elliott P, Frenneaux MP. Biventricular pacemaker therapy improves exercise capacity in patients with non-obstructive hypertrophic cardiomyopathy via augmented diastolic filling on exercise. Eur J Heart Fail. 2020 Jul;22(7):1263-1272. doi: 10.1002/ejhf.1722. Epub 2020 Jan 23. PMID: 31975494; PMCID: PMC7540697.

[36] Stockburger, M., Moss, A.J., Klein, H.U. *et al.* **S**ustained clinical benefit of cardiac resynchronization therapy in non-LBBB patients with prolonged PR-interval: MADIT-CRT long-term follow-up. *Clin Res Cardiol* **105**, 944–952 (2016). https://doi.org/10.1007/s00392-016-1003-z

[37] Goldenberg I, Moss AJ, Hall WJ, Foster E, Goldberger JJ, Santucci P, Shinn T, Solomon S, Steinberg JS, Wilber D, Barsheshet A, McNitt S, Zareba W, Klein H; MADIT-CRT Executive Committee. Predictors of response to cardiac resynchronization therapy in the Multicenter Automatic Defibrillator Implantation Trial with Cardiac Resynchronization Therapy (MADIT-CRT). Circulation. 2011 Oct 4;124(14):1527-36. doi: 10.1161/CIRCULATIONAHA.110.014324. Epub 2011 Sep 6. PMID: 21900084.

[38] Cleland JG, Freemantle N, Erdmann E, Gras D, Kappenberger L, Tavazzi L, Daubert JC. Long-term mortality with cardiac resynchronization therapy in the Cardiac Resynchronization-Heart Failure (CARE-HF) trial. Eur J Heart Fail. 2012 Jun;14(6):628-34. doi: 10.1093/eurjhf/hfs055. Epub 2012 May 2. PMID: 22552183.

[39] Gillis AM, Kerr CR, Philippon F, Newton G, Talajic M, Froeschl M, Froeschl S, Swiggum E, Yetisir E, Wells GA, Tang AS. Impact of cardiac resynchronization therapy on hospitalizations in the Resynchronization-Defibrillation for Ambulatory Heart Failure trial. Circulation. 2014 May 20;129(20):2021-30. doi: 10.1161/CIRCULATIONAHA.112.000417. Epub 2014 Mar 7. PMID: 24610807.

[40] Hsu JC, Solomon SD, Bourgoun M, McNitt S, Goldenberg I, Klein H, Moss AJ, Foster E; MADIT-CRT Executive Committee. Predictors of super-response to cardiac resynchronization therapy and associated improvement in clinical outcome: the MADIT-CRT (multicenter automatic defibrillator implantation trial with cardiac resynchronization therapy) study. J Am Coll Cardiol. 2012 Jun 19;59(25):2366-73. doi: 10.1016/j.jacc.2012.01.065. PMID: 22698490.

[41] Gold MR, Daubert C, Abraham WT, Ghio S, St John Sutton M, Hudnall JH, Cerkvenik J, Linde C. The effect of reverse remodeling on long-term survival in mildly symptomatic patients with heart failure receiving cardiac resynchronization therapy: results of the REVERSE study. Heart Rhythm. 2015 Mar;12(3):524-530. doi: 10.1016/j.hrthm.2014.11.014. Epub 2014 Nov 15. PMID: 25460860; PMCID: PMC4390984.

[42] Zhang Y, Xing Q, Zhang JH, Jiang WF, Qin M, Liu X. Long-Term Effect of Different Optimizing Methods for Cardiac Resynchronization Therapy in Patients with Heart Failure: A Randomized and Controlled Pilot Study. Cardiology. 2019;142(3):158-166. doi: 10.1159/000499502. Epub 2019 Jun 12. PMID: 31189165.

[43] Barsheshet A, Goldenberg I, Moss AJ, Eldar M, Huang DT, McNitt S, Klein HU, Hall WJ, Brown MW, Goldberger JJ, Goldstein RE, Schuger C, Zareba W, Daubert JP. Response to preventive cardiac resynchronization therapy in patients with ischaemic and nonischaemic cardiomyopathy in MADIT-CRT. Eur Heart J. 2011 Jul;32(13):1622-30. doi: 10.1093/eurheartj/ehq407. Epub 2010 Nov 12. PMID: 21075774.

[44] Biton Y, Costa J, Zareba W, Baman JR, Goldenberg I, McNitt S, Solomon SD, Polonsky B, Kutyifa V. Predictors of long-term mortality with cardiac resynchronization therapy in mild heart failure patients with left bundle branch block. Clin Cardiol. 2018 Oct;41(10):1358-1366. doi: 10.1002/clc.23058. PMID: 30141210; PMCID: PMC6490024.

[45] Gasparini M, Leclercq C, Yu CM, Auricchio A, Steinberg JS, Lamp B, Klersy C, Leyva F. Absolute survival after cardiac resynchronization therapy according to baseline QRS duration: a multinational 10-year experience: data from the Multicenter International CRT Study. Am Heart J. 2014 Feb;167(2):203-209.e1. doi: 10.1016/j.ahj.2013.10.017. Epub 2013 Nov 4. PMID: 24439981.

[46] Schuchert A, Muto C, Maounis T, Ella RO, Polauck A, Padeletti L; MASCOT study group. Relationship between pre-implant ejection fraction and outcome after cardiac resynchronization therapy in symptomatic patients. Acta Cardiol. 2014 Aug;69(4):424-32. doi: 10.1080/ac.69.4.3036659. PMID: 25181918.

[47] Kristiansen HM, Vollan G, Hovstad T, Keilegavlen H, Faerestrand S. A randomized study of haemodynamic effects and left ventricular dyssynchrony in right ventricular apical vs. high posterior septal pacing in cardiac resynchronization therapy. Eur J Heart Fail. 2012 May;14(5):506-16. doi: 10.1093/eurjhf/hfr162. Epub 2012 Jan 26. PMID: 22286156.

[48] Yu CM, Fang F, Zhang Q, Yip GW, Li CM, Chan JY, Wu L, Fung JW. Improvement of atrial function and atrial reverse remodeling after cardiac resynchronization therapy for heart failure. J Am Coll Cardiol. 2007 Aug 21;50(8):778-85. doi: 10.1016/j.jacc.2007.04.073. Epub 2007 Aug 6. PMID: 17707183.

[49] Schuchert A, Muto C, Maounis T, Frank R, Ella RO, Polauck A, Padeletti L; Mascot Study Group. One-year outcome after CRT implantation in NYHA class IV in comparison to NYHA class III patients. Clin Res Cardiol. 2013 Jul;102(7):505-11. doi: 10.1007/s00392-013-0558-1. Epub 2013 Mar 31. PMID: 23543129.

[50] Diab IG, Hunter RJ, Kamdar R, Berriman T, Duncan E, Richmond L, Baker V, Abrams D, Earley MJ, Sporton S, Schilling RJ. Does ventricular dyssynchrony on echocardiography predict response to cardiac resynchronisation therapy? A randomised controlled study. Heart. 2011 Sep;97(17):1410-6. doi: 10.1136/hrt.2011.227686. Epub 2011 Jun 23. PMID: 21700757.

[51] J. Walmsley, WM. Van Everdingen, MJM Cramer, T. Delhaas, FW. Prinzen, GE. Leenders, J. Lumens, P249
Right ventricular function modulates acute response to cardiac resynchronization therapy, *EP Europace*, Volume 19, Issue suppl_3, June 2017, Pages iii30–iii31, https://doi.org/10.1093/ehjci/eux171.006

[52] Kalscheur MM, Saxon LA, Lee BK, Steinberg JS, Mei C, Buhr KA, DeMets DL, Bristow MR, Singh SN. Outcomes of cardiac resynchronization therapy in patients with intermittent atrial fibrillation or atrial flutter in the COMPANION trial. Heart Rhythm. 2017 Jun;14(6):858-865. doi: 10.1016/j.hrthm.2017.03.024. Epub 2017 Mar 18. PMID: 28323173.

[53] Asbach S, Lennerz C, Semmler V, Grebmer C, Solzbach U, Kloppe A, Klein N, Szendey I, Andrikopoulos G, Tzeis S, Bode C, Kolb C; SPICE Study Investigators. Impact of the Right Ventricular Lead Position on Clinical End Points in CRT Recipients--A Subanalysis of the Multicenter Randomized SPICE Trial. Pacing Clin Electrophysiol. 2016 Mar;39(3):261-7. doi: 10.1111/pace.12793. Epub 2016 Jan 12. PMID: 26643821.

[54] Ricci F, Mele D, Bianco F, Bucciarelli V, De Caterina R, Gallina S. Right heart-pulmonary circulation unit and cardiac resynchronization therapy. Am Heart J. 2017 Mar;185:1-16. doi: 10.1016/j.ahj.2016.11.005. Epub 2016 Nov 17. PMID: 28267462.

[55] Sharma A, Lavie CJ, Vallakati A, Garg A, Goel S, Lazar J, Fonarow GC. Changes in parameters of right ventricular function with cardiac resynchronization therapy. Clin Cardiol. 2017 Nov;40(11):1033-1043. doi: 10.1002/clc.22762. Epub 2017 Sep 12. PMID: 28898433; PMCID: PMC6490373.

[56] Sharma A, Bax JJ, Vallakati A, Goel S, Lavie CJ, Garg A, Mukherjee D, Lichstein E, Lazar JM. Effect of cardiac resynchronization therapy on right ventricular function. Int J Cardiol. 2016 Apr 15;209:34-6. doi: 10.1016/j.ijcard.2016.02.034. Epub 2016 Feb 4. PMID: 26878471.

[57] Sharma A, Bax JJ, Vallakati A, Goel S, Lavie CJ, Kassotis J, Mukherjee D, Einstein A, Warrier N, Lazar JM. Meta-Analysis of the Relation of Baseline Right Ventricular Function to Response to Cardiac Resynchronization Therapy. Am J Cardiol. 2016 Apr 15;117(8):1315-21. doi: 10.1016/j.amjcard.2016.01.029. Epub 2016 Jan 28. PMID: 26879068.

Table S5. Quality assessment of included studies (Newcastle-Ottawa scale).

	Cohort studies								
	**Selection**				**Comparability**	**Exposure**			
Study	Representativeness of cohort	Selection of the non-exposed cohort	Ascerainement of exposure	Outcome of interest was not present at start of study		Assessmment of outcomes	Length of follow-up	Adequacy of follow-up	**Total**
Plata-corona 2024	*	-	*	*	*	*	*	*	******* (7)
Dawood2023	*	-	*	*	*	*	*	*	*******(7)
Yuecel2023	*	-	*	*	*	*	*	*	*******(7)
Topal2023	*	-	*	*	*	*	*	*	*******(7)
Sadeghian2022	*	-	*	*	-*	*	*	*	*******(7)
Deaconu2021	*	-	*	*	**	*	*	*	********(8)
Cruz2019	*	-	*	*	*	*	*	*	*******(7)
Braganca2019	*	-	*	*	**	*	*	*	*********(9)
Martens2018	*	-	*	*	*	*	*	*	*******(7)
Abdelhamid 2017	*	-	*	*	**	*	*	*	********(8)
Park2016	*	-	*	*	*	*	*	*	*******(7)
Rapaciuolo2016	*	-	*	*	*	*	*	*	*******(7)
Kang2015	*	-	*	*	*	*	*	*	*******(7)
Zaborska2014	*	-	*	*	*	*	*	*	*******(7)
Luca2014	*	-	*	*	*	*	*	*	*******(7)
Knappe2013	*	-	*	*	*	*	*	*	*******(7)
Leong2013	*	-	*	*	*	*	*	*	*******(7)
Sade2013	*	-	*	*	*	*	*	*	*******(7)
Praus2012	*	-	*	*	*	*	*	*	*******(7)
Kusiak2012	*	-	*	*	*	*	*	*	*******(7)
Szulik2011	*	-	*	*	*	*	*	*	*******(7)
Vitarelli2011	*	-	*	*	*	*	*	*	*******(7)
Aksoy2011	*	-	*	*	*	*	*	*	*******(7)
Esmaelzadeh 2011	*	-	*	*	*	*	-	*	******(6)
Anter2010	*	-	*	*	*	-	*	-	*****(5)
DAndrea2009	*	-	*	*	*	*	*	*	*******(7)
Scuteri2009	*	-	*	*	**	*	*	*	********(8)
Yuasa2009	*	-	*	*	*	*	*	*	*******(7)
Donal2008	*	-	*	*	*	*	*	*	*******(7)
Bleeker2005	*	-	*	*	*	*	*	*	*******(7)

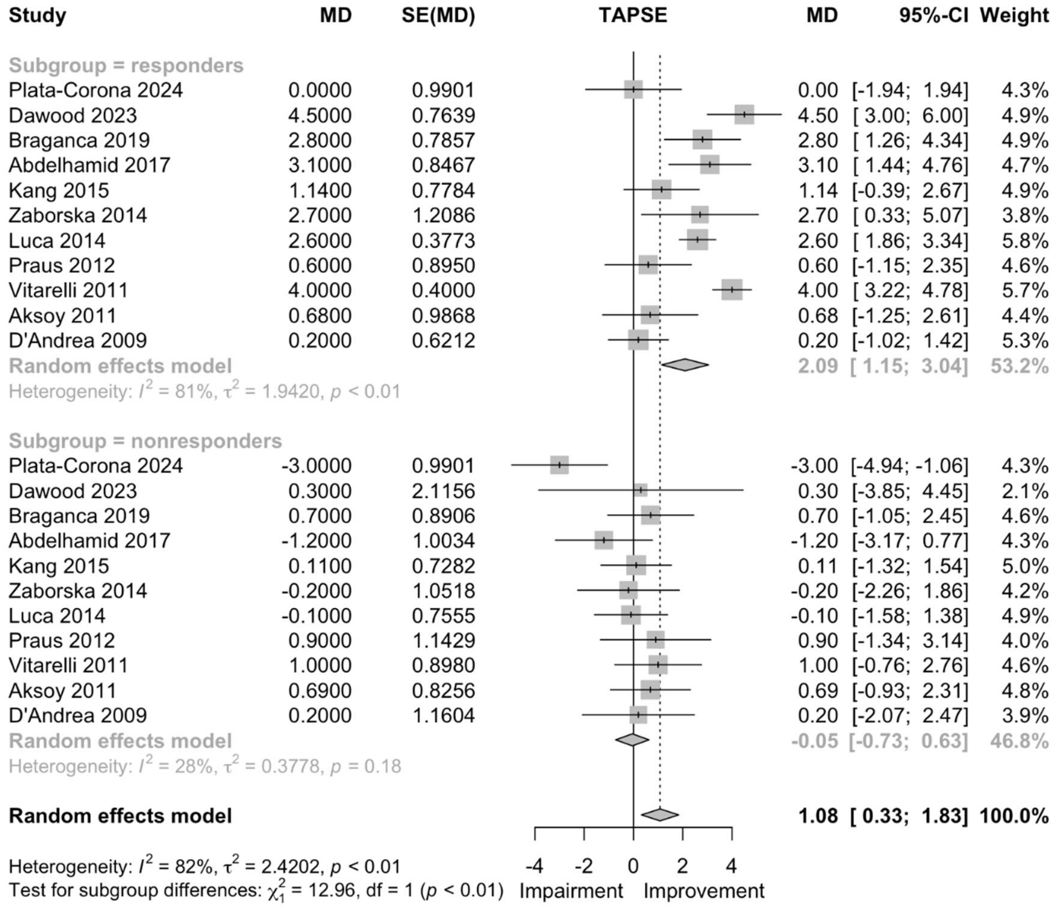

Figure S2. Subgroup analysis of tricuspid annular plane systolic excursion (TAPSE) based on the response to cardiac resynchronization therapy. Abbreviations: CI, confidence interval; MD, mean difference; SE, standard error.

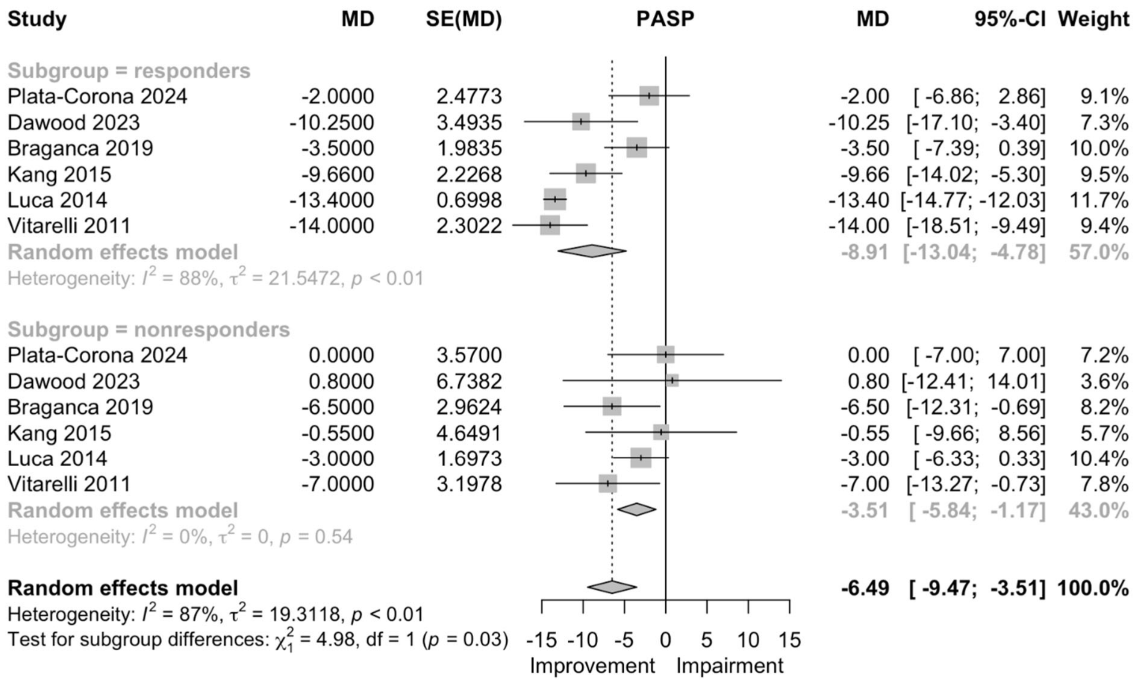

Figure S3. Subgroup analysis of pulmonary artery systolic pressure (PASP) based on the response to cardiac resynchronization therapy. Abbreviations: CI, confidence interval; MD, mean difference; SE, standard error.

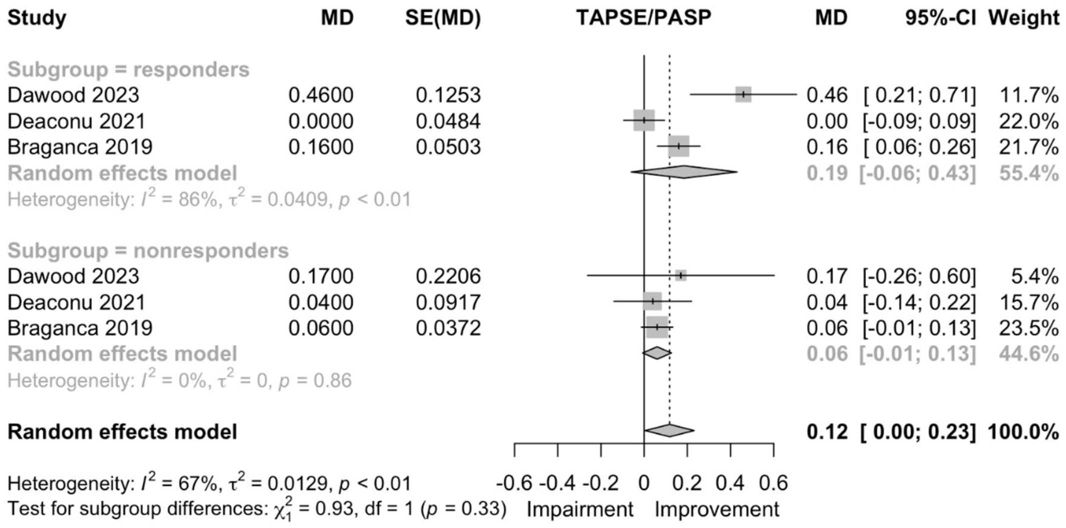

Figure S4. Subgroup analysis of TAPSE/PASP ratio based on the response to cardiac resynchronization therapy. Abbreviations: CI, confidence interval; MD, mean difference; SE, standard error.

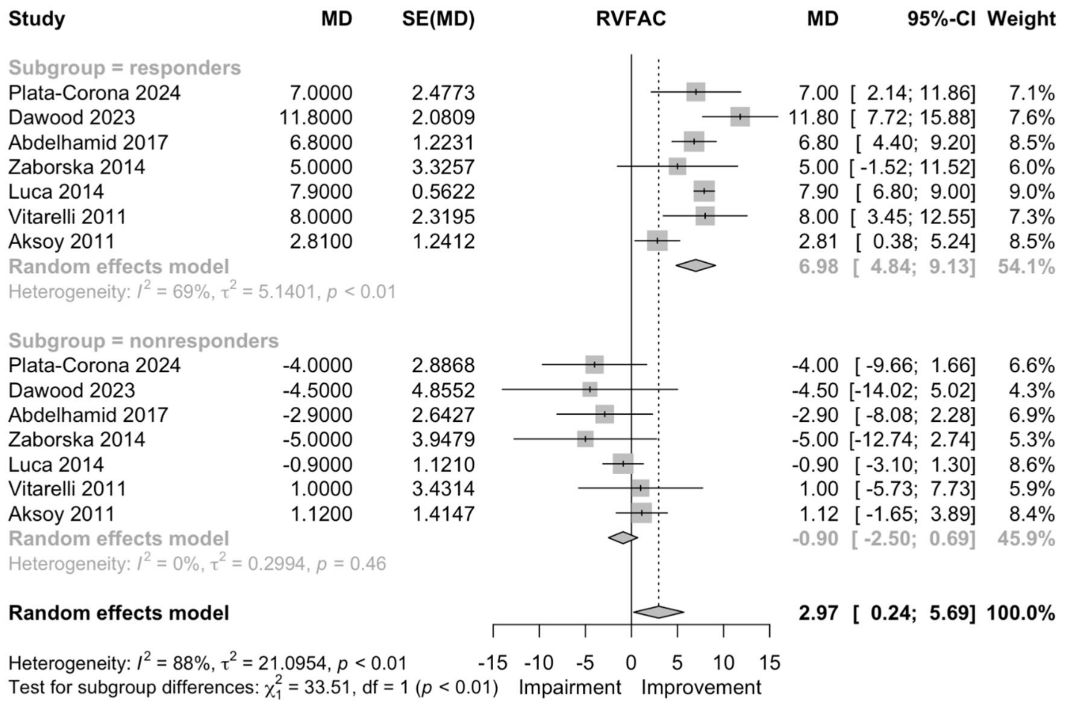

Figure S5. Subgroup analysis of right ventricular fractional shortening (RVFAC) based on the response to cardiac resynchronization therapy. Abbreviations: CI, confidence interval; MD, mean difference; SE, standard error.

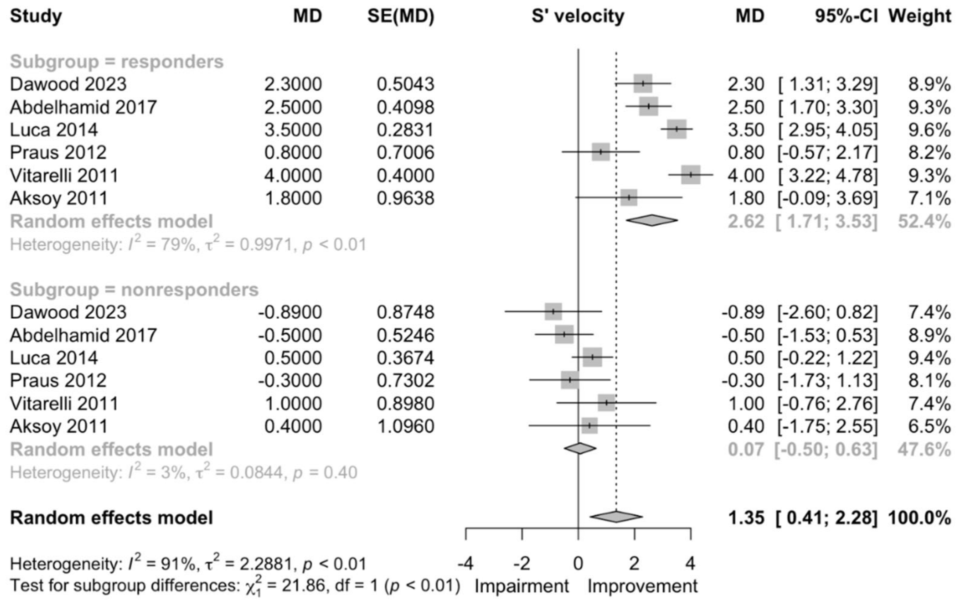

Figure S6. Subgroup analysis of tricuspid annular systolic velocity (S′ velocity) based on the response to cardiac resynchronization therapy. Abbreviations: CI, confidence interval; MD, mean difference; SE, standard error.

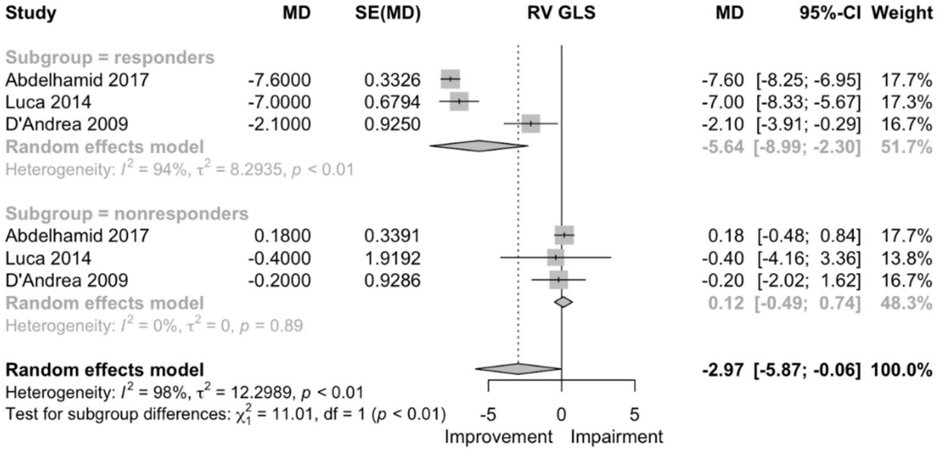

Figure S7. Subgroup analysis of right ventricular longitudinal strain (RV GLS) based on the response to cardiac resynchronization therapy. Abbreviations: CI, confidence interval; MD, mean difference; SE, standard error.

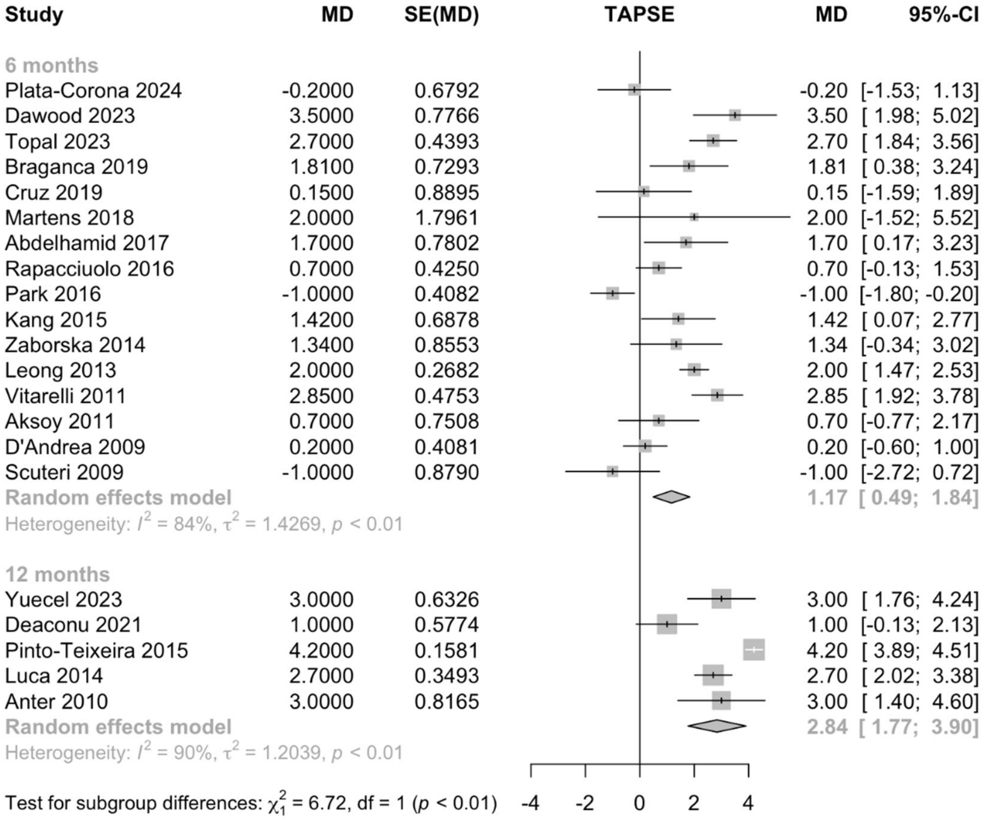

Figure S8. Subgroup analysis of tricuspid annular plane systolic excursion (TAPSE) at 6 and 12 months. Abbreviations: CI, confidence interval; MD, mean difference; SE, standard error.

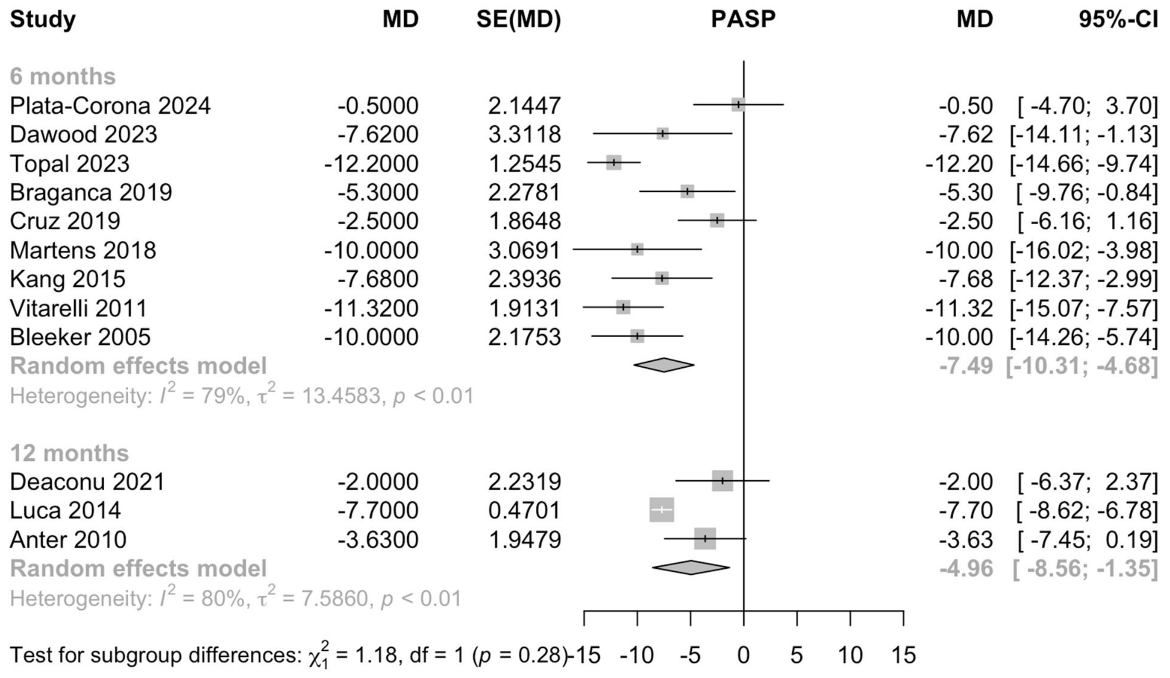

Figure S9. Subgroup analysis of pulmonary artery systolic pressure (PASP) at 6 and 12 months. Abbreviations: CI, confidence interval; MD, mean difference; SE, standard error.

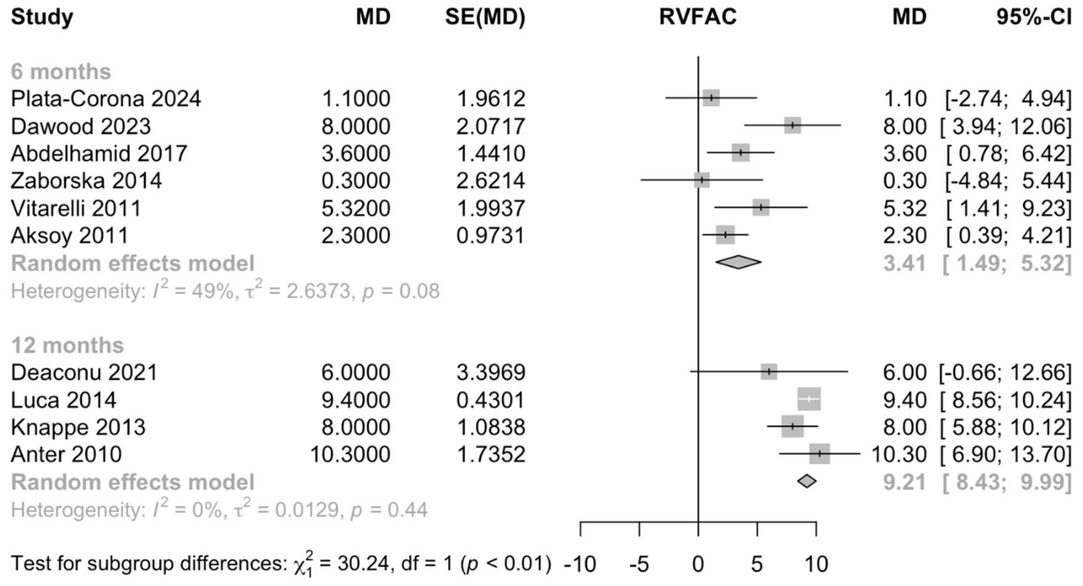

Figure S10. Subgroup analysis of right ventricular fractional shortening (RVFAC) at 6 and 12 months. Abbreviations: CI, confidence interval; MD, mean difference; SE, standard error.

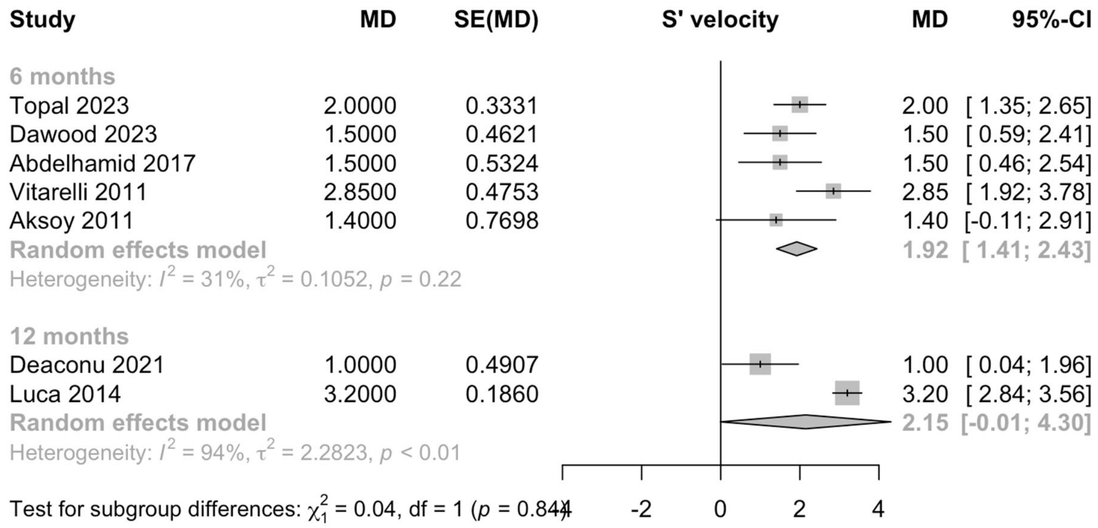

Figure S11. Subgroup analysis of tricuspid annular systolic velocity (S′ velocity) at 6 and 12 months. Abbreviations: CI, confidence interval; MD, mean difference; SE, standard error.

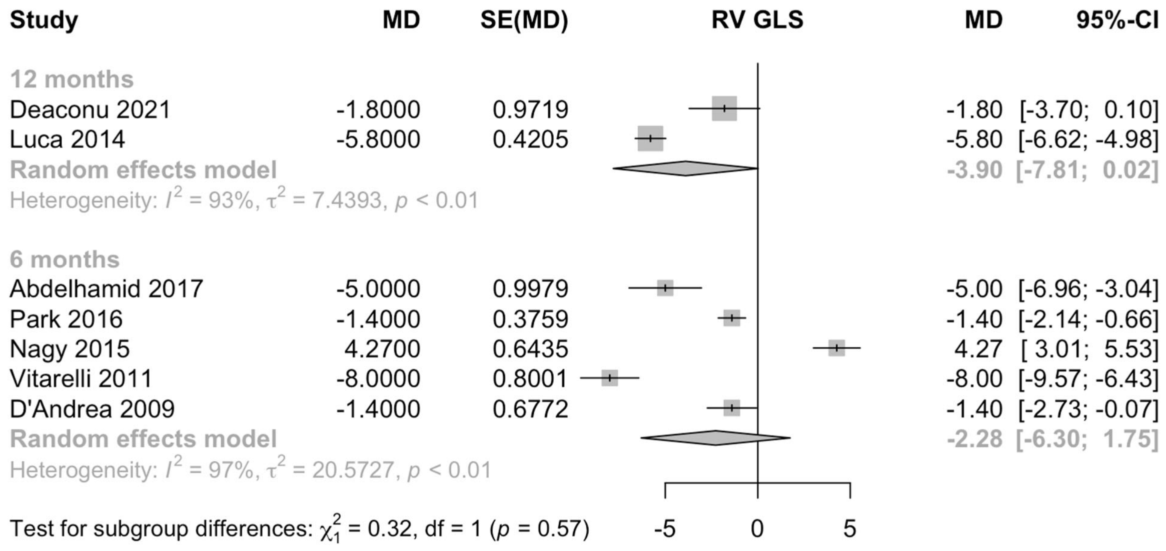

Figure S12. Subgroup analysis of right ventricular longitudinal strain (RV GLS) at 6 and 12 months. Abbreviations: CI, confidence interval; MD, mean difference; SE, standard error.

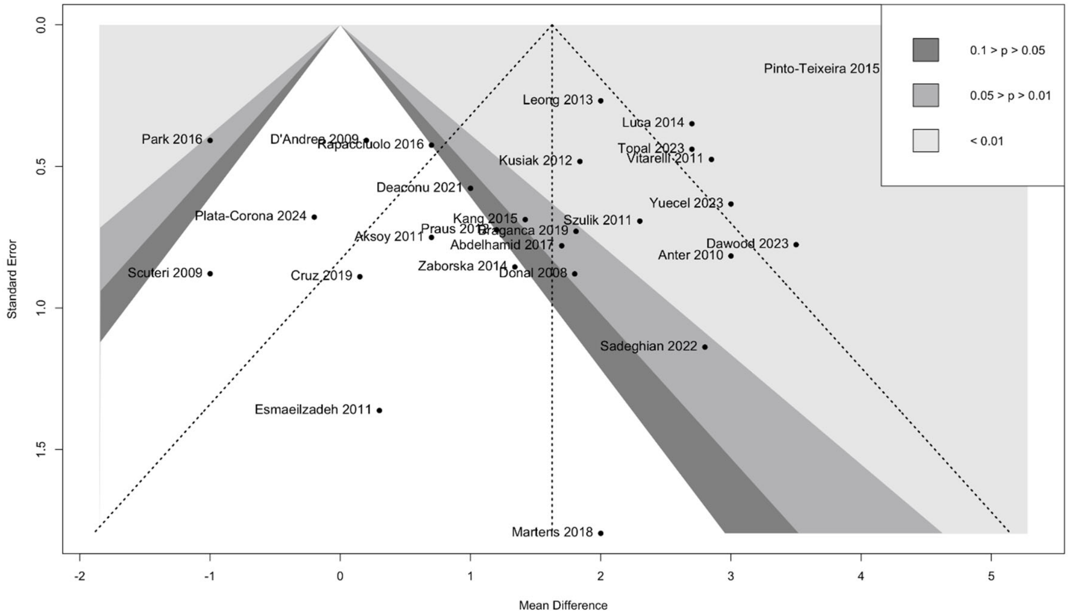

Figure S13. Contour- enhanced funnel plot of effect size versus standard error for the association between cardiac resynchronization therapy and change in tricuspid annular plane systolic excursion (TAPSE).

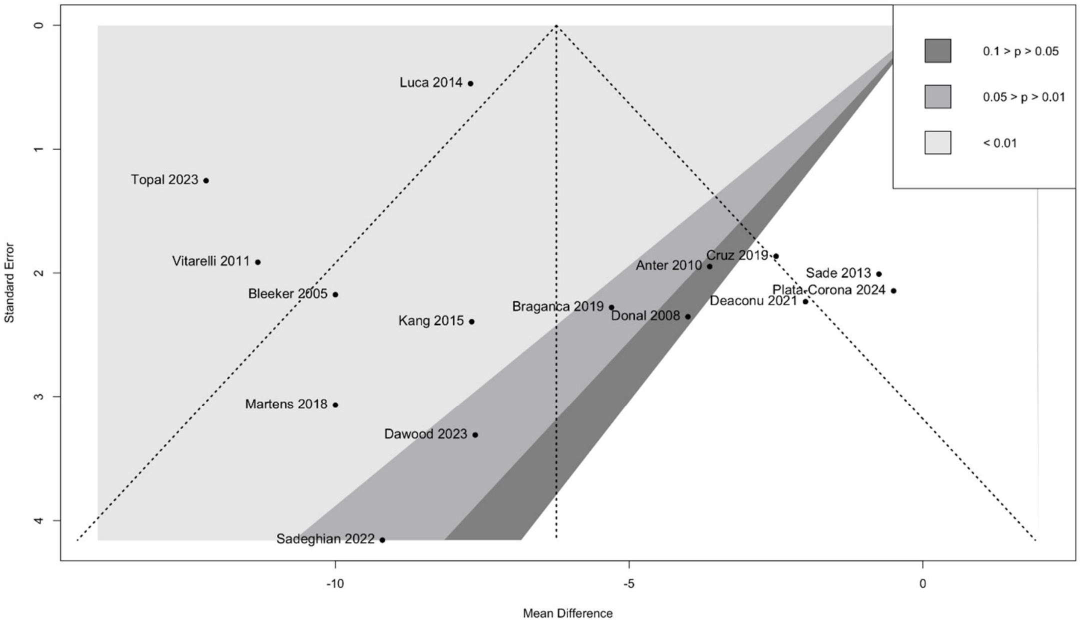

Figure S14. Contour- enhanced funnel plot of effect size versus standard error for the association between cardiac resynchronization therapy and change in pulmonary artery systolic pressure (PASP).

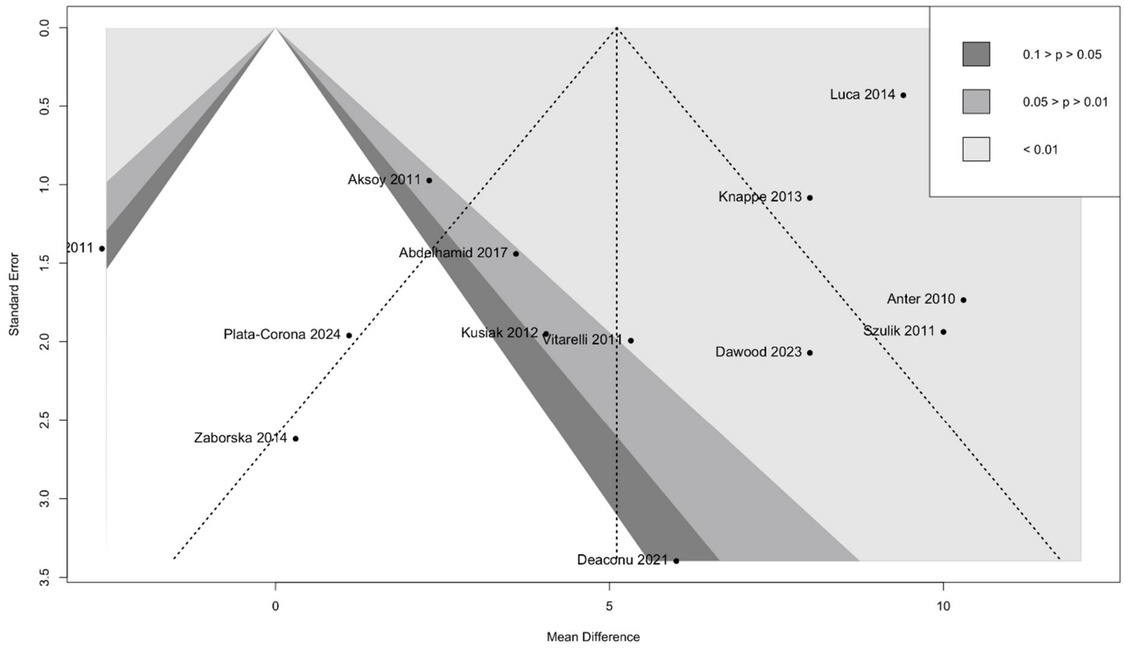

Figure S15. Contour- enhanced funnel plot of effect size versus standard error for the association between cardiac resynchronization therapy and change in right ventricular fractional shortening (RVFAC).

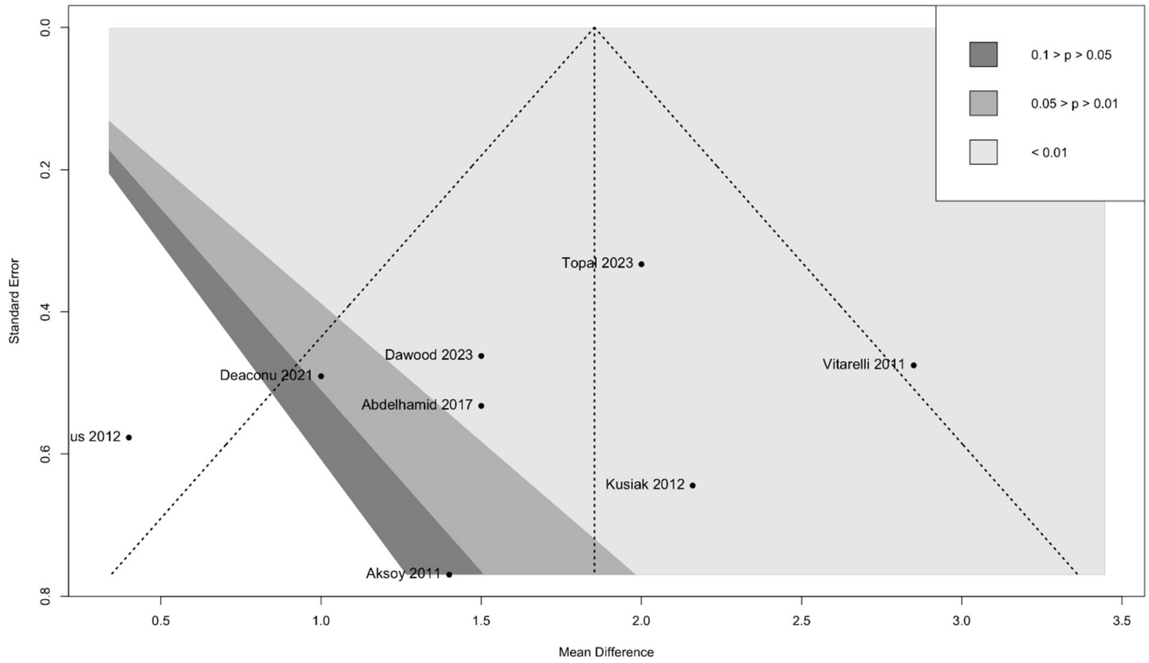

Figure S16. Contour- enhanced funnel plot of effect size versus standard error for the association between cardiac resynchronization therapy and change in tricuspid annular systolic velocity (S′ velocity).

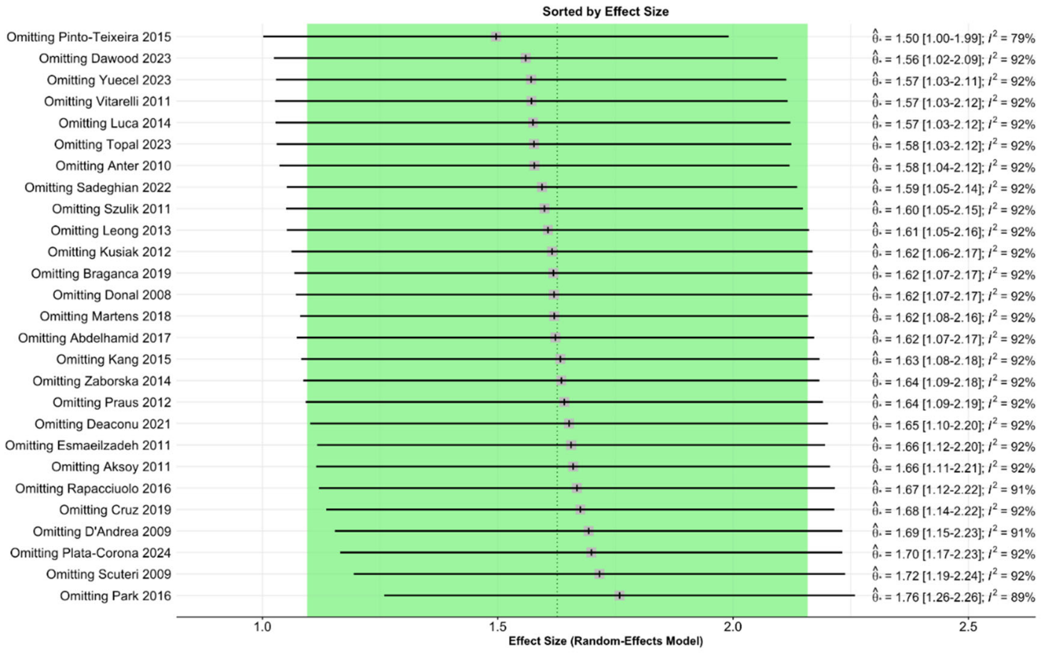

Figure S17. Leave-one-out sensitivity analysis sorted by effect size on the association cardiac between cardiac resynchronization therapy and change in tricuspid annular plane systolic excursion (TAPSE).

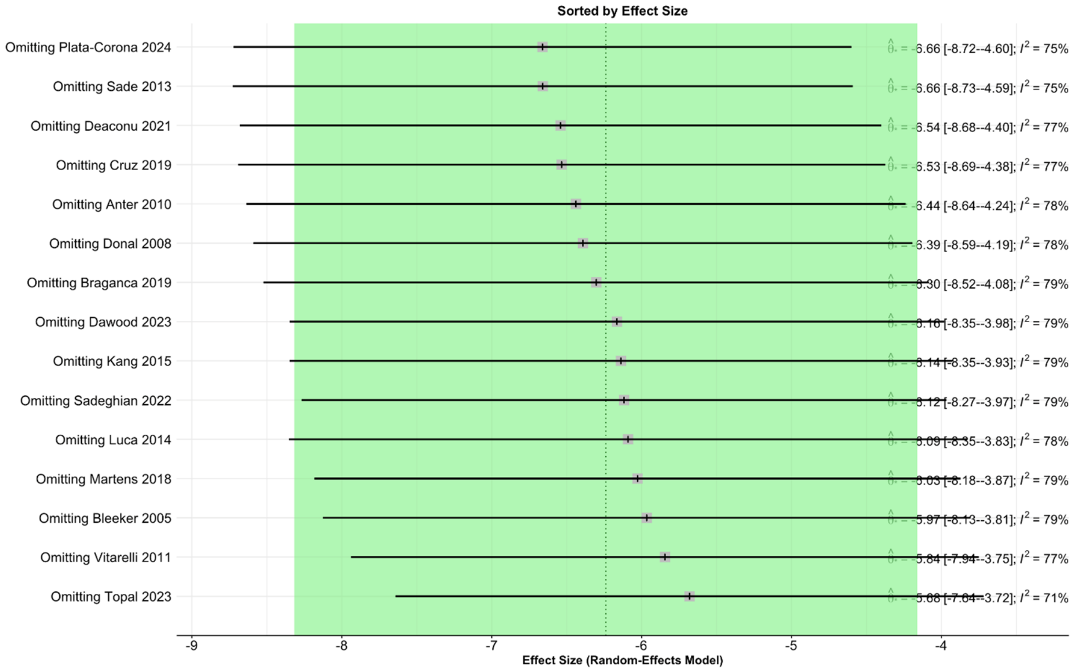

Figure S18. Leave-one-out sensitivity analysis sorted by effect size on the association cardiac between cardiac resynchronization therapy and change in change in pulmonary artery systolic pressure (PASP).

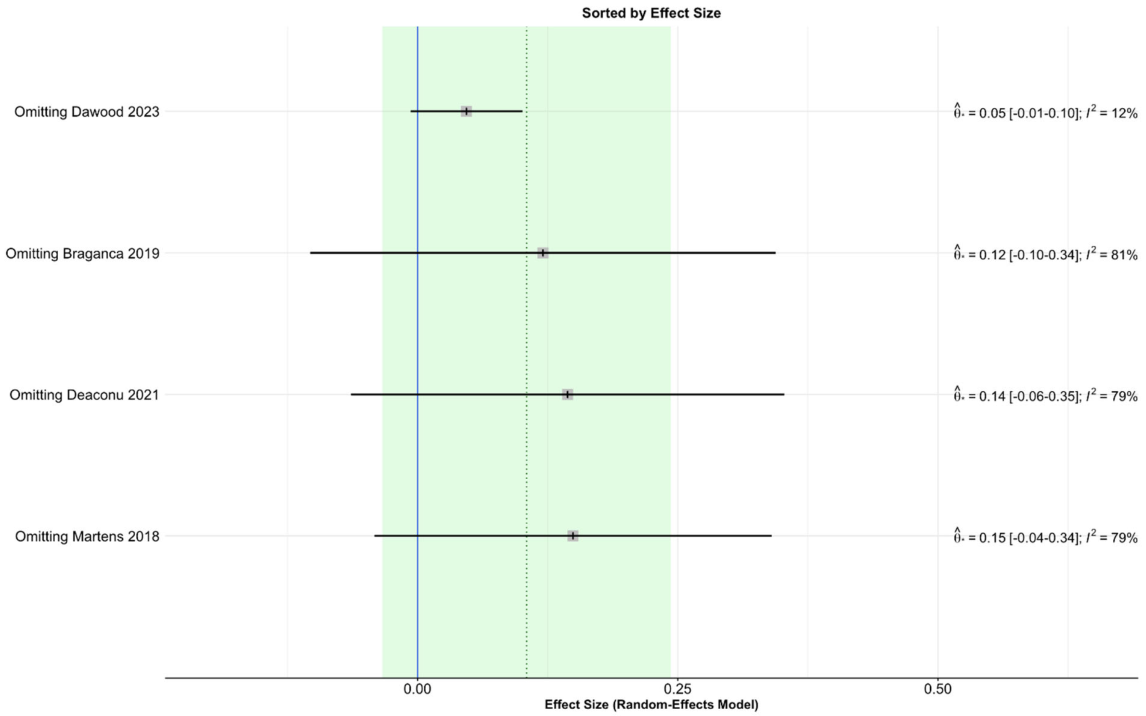

Figure S19. Leave-one-out sensitivity analysis sorted by effect size on the association cardiac between cardiac resynchronization therapy and change in change in TAPSE/PASP ratio.

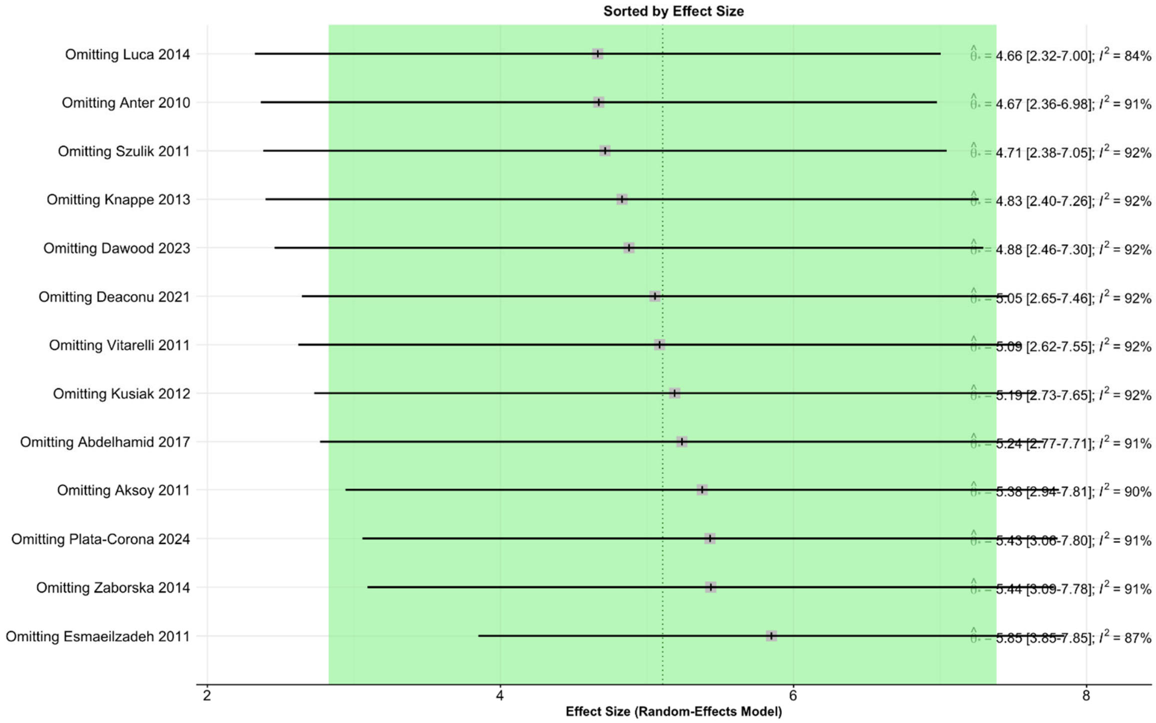

Figure S20. Leave-one-out sensitivity analysis sorted by effect size on the association cardiac between cardiac resynchronization therapy and change in right ventricular fractional shortening (RVFAC).

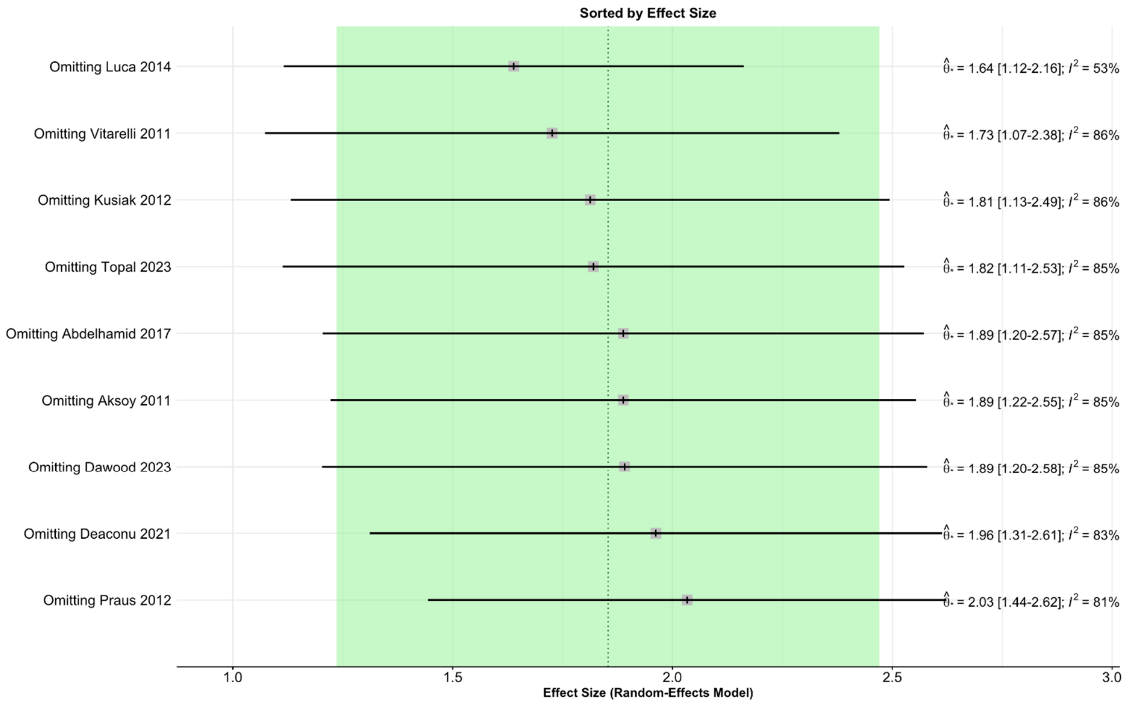

Figure S21. Leave-one-out sensitivity analysis sorted by effect size on the association cardiac between cardiac resynchronization therapy and change in tricuspid annular systolic velocity (S' velocity).

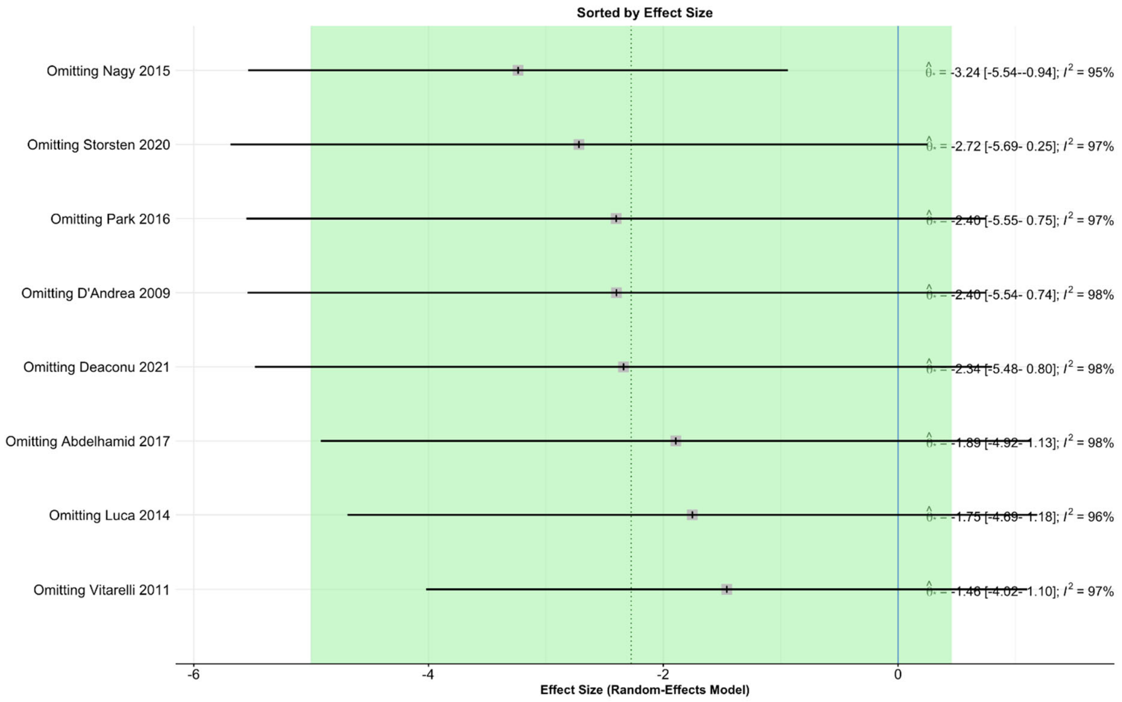

Figure S22. Leave-one-out sensitivity analysis sorted by effect size on the association cardiac between cardiac resynchronization therapy and change in right ventricular longitudinal strain (RV GLS).

Table S6. Meta-regression analysis on tricuspid annular plane systolic excursion (TAPSE)

Covariate	No of studies	β	P value†	Confidence interval	tau²	R² ‡	I² ˠ
TAPSE							
Publication year	27	0.0426	0.4557	-0.0693 to 0.1544	1.5323	0%	86.19%
Follow-up (months)	27	0.1555	0.0186*	0.0260 to 0.2849	1.2173	18.44%	81.61%
Age (years)	27	0.0498	0.3761	-0.0605 to 0.1601	1.5068	0%	85.74%
Male (%)	27	0.0510	0.0424*	0.0018 to 0.1003	1.2710	14.85%	83.21%
Baseline LVEF (%)	25	0.0651	0.5174	-0.1320 to 0.2611	1.5790	0%	86.25%
Baseline LVESV (ml)	16	0.0013	0.8858	-0.0162 to 0.0188	0.7420	0%	67.44%
Baseline LVEDV (ml)	13	-0.0018	0.8105	-0.0169 to 0.0132	0.8823	0%	62.64%
Baseline QRS (ms)	21	0.0426	0.0787	-0.0049 to 0.0900	1.4344	14.70%	82.41%
Baseline NYHA II-III (%)	15	-0.0054	0.7306	-0.0365 to 0.0256	0.7516	0%	69.92%
Ischemic cardiomyopathy (%)	26	0.0148	0.3625	-0.0170 to 0.0466	1.5098	0%	85.68%
Diabetes (%)	15	0.0104	0.7479	-0.0531 to 0.0739	0.7447	0%	66.87%
Hypertension (%)	14	-0.0127	0.4909	-0.0490 to 0.0235	0.8605	0%	67.38%
Use of ACEI/ARBs (%)	15	-0.0126	0.8245	-0.1237 to 0.0986	1.3524	0%	80.64%
Use of beta-blockers (%)	15	0.0045	0.9189	-0.0818 to 0.0907	1.3669	0%	78.89%
Use of diuretics (%)	14	-0.0525	0.2800	-0.1477 to 0.0427	1.1969	0%	78.33%

Abbreviations: LVEF, left ventricular ejection fraction; LVEDV, left ventricular end-diastolic volume; LVESV, left ventricular end-systolic volume; NYHA, New York Heart Association classification. † P-value from a Wald test for the effect of the covariate in the model. ‡ R^2, the relative reduction in the between-study variance ˠ I^2, the percentage of the residual variation that is attributable to between-study heterogeneity

Table S7. Meta-regression analysis on right ventricular fractional shortening (RVFAC).

Covariate	No of studies	β	P value†	Confidence interval	tau²	R² ‡	I² ˠ
RVFAC							
Publication year	13	-0.0638	0.8084	-0.5797 to 0.4521	15.6234	0%	90.36%
Follow-up (months)	13	0.7656	<0.0001***	0.5135 to 1.0176	1.8376	87.15%	48.66%
Age (years)	13	0.1838	0.5124	-0.3661 to 0.7337	14.9058	0%	89.37%
Male (%)	13	0.0705	0.5034	-0.1359 to 0.2768	14.7250	0%	89.01%
Baseline LVEF (%)	12	0.7735	0.0061**	0.2205 to 1.3265	7.5756	46.31%	78.54%
Baseline LVESV (ml)	8	0.0544	0.2397	-0.0363 to 0.1451	12.8707	6.46%	83.89%
Baseline LVEDV (ml)	7	0.0550	0.1001	-0.0106 to 0.1205	11.3788	25.25%	83.96%
Baseline QRS (ms)	9	0.1795	0.0409*	0.0074 to 0.3515	8.6768	35.72%	78.13%
Baseline NYHA II-III (%)	7	-0.2360	0.0137*	-0.4237 to -0.0484	3.6274	62.42%	53.76%
Ischemic cardiomyopathy (%)	12	-0.0213	0.7394	-0.1471 to 0.1044	14.8483	0%	87.85%
Diabetes (%)	6	-0.0515	0.8103	-0.4717 to 0.3688	11.4780	0%	78.43%
Hypertension (%)	6	-0.1167	0.1824	-0.2833 to 0.0548	6.6753	25.7%	62.81%
Use of ACEI/ARBs (%)	6	-0.3230	0.0141*	-0.5808 to -0.0652	1.7617	72.94%	32.88%

Abbreviations: LVEF, left ventricular ejection fraction; LVEDV, left ventricular end-diastolic volume; LVESV, left ventricular end-systolic volume; NYHA, New York Heart Association classification. † P-value from a Wald test for the effect of the covariate in the model. ‡ R^2, the relative reduction in the between-study variance. ˠ I^2, the percentage of the residual variation that is attributable to between-study heterogeneity

Table S8. Meta-regression analysis on pulmonary artery systolic pressure (PASP)

Covariate	No of studies	β	P value†	Confidence interval	tau^2	R^2 ‡	I^2 ˠ
PASP							
Publication year	15	0.0624	0.7423	-0.3097 to 0.4345	12.9346	0%	81.22%
Follow-up (months)	15	0.2205	0.1290	-0.0642 to 0.5052	10.3918	12.70%	77.99%
Age (years)	15	-0.1587	0.6105	-0.7692 to 0.4519	12.6876	0%	81.72%
Male (%)	15	-0.1362	0.2508	-0.3687 to 0.0963	11.5709	2.80%	76.70%
Baseline LVEF (%)	15	0.0995	0.7528	-0.5198 to 0.7188	12.9415	0%	77.38%
Baseline LVESV (ml)	8	-0.0859	0.0328*	-0.1648 to -0.0070	7.6586	49.70%	60.11%
Baseline LVEDV (ml)	6	-0.0628	0.0280*	-0.1187 to -0.0068	2.4534	76.42%	25.66%
Baseline QRS (ms)	9	-0.0049	0.9799	-0.3870 to 0.3772	10.2349	0%	62.97%
Baseline NYHA II-III (%)	9	-00117	0.9295	-0.2711 to 0.2477	16.1436	0%	78.02%
Ischemic cardiomyopathy (%)	14	-0.0768	0.1789	-0.1887 to 0.0352	11.1113	11.43%	74.88%
Diabetes (%)	9	0.0690	0.6726	-0.2509 to 0.3888	15.4114	0%	75.88%
Hypertension (%)	9	0.0113	0.8859	-0.1433 to 0.1659	15.8058	0%	78.96%
Use of ACEI/ARBs (%)	7	-0.5600	0.4569	-2.0353 to 0.9153	12.2176	0%	68.62%
Use of beta-blockers (%)	8	0.1548	0.0757	-0.0160 to 0.3255	5.4977	40.28%	49.66%
Use of diuretics (%)	6	0.3520	0.0061*	0.1004 to 0.6035	0	100%	0%

Abbreviations: LVEF, left ventricular ejection fraction; LVEDV, left ventricular end-diastolic volume; LVESV, left ventricular end-systolic volume; NYHA, New York Heart Association classification. † P-value from a Wald test for the effect of the covariate in the model. ‡ R^2, the relative reduction in the between-study variance. ˠ I^2, the percentage of the residual variation that is attributable to between-study heterogeneity.

Table S9. PRISMA 2020 Checklist

Section and Topic	Item #	Checklist item	Location where item is reported
**TITLE**			
Title	1	Identify the report as a systematic review.	Page 1
**ABSTRACT**			
Abstract	2	See the PRISMA 2020 for Abstracts checklist.	Done
**INTRODUCTION**			
Rationale	3	Describe the rationale for the review in the context of existing knowledge.	Page 6
Objectives	4	Provide an explicit statement of the objective(s) or question(s) the review addresses.	Page 6
**METHODS**			
Eligibility criteria	5	Specify the inclusion and exclusion criteria for the review and how studies were grouped for the syntheses.	Page 7
Information sources	6	Specify all databases, registers, websites, organisations, reference lists and other sources searched or consulted to identify studies. Specify the date when each source was last searched or consulted.	Pages 6-7
Search strategy	7	Present the full search strategies for all databases, registers and websites, including any filters and limits used.	Sup. Tables 1-3
Selection process	8	Specify the methods used to decide whether a study met the inclusion criteria of the review, including how many reviewers screened each record and each report retrieved, whether they worked independently, and if applicable, details of automation tools used in the process.	Page 6
Data collection process	9	Specify the methods used to collect data from reports, including how many reviewers collected data from each report, whether they worked independently, any processes for obtaining or confirming data from study investigators, and if applicable, details of automation tools used in the process.	Page 7
Data items	10a	List and define all outcomes for which data were sought. Specify whether all results that were compatible with each outcome domain in each study were sought (e.g. for all measures, time points, analyses), and if not, the methods used to decide which results to collect.	Page 7
	10b	List and define all other variables for which data were sought (e.g. participant and intervention characteristics, funding sources). Describe any assumptions made about any missing or unclear information.	Page 7
Study risk of bias assessment	11	Specify the methods used to assess risk of bias in the included studies, including details of the tool(s) used, how many reviewers assessed each study and whether they worked independently, and if applicable, details of automation tools used in the process.	Page 8
Effect measures	12	Specify for each outcome the effect measure(s) (e.g. risk ratio, mean difference) used in the synthesis or presentation of results.	Page 7-8
Synthesis methods	13a	Describe the processes used to decide which studies were eligible for each synthesis (e.g. tabulating the study intervention characteristics and comparing against the planned groups for each synthesis (item #5)).	Page 8
	13b	Describe any methods required to prepare the data for presentation or synthesis, such as handling of missing summary statistics, or data conversions.	Page 7
	13c	Describe any methods used to tabulate or visually display results of individual studies and syntheses.	Tables 1-2
	13d	Describe any methods used to synthesize results and provide a rationale for the choice(s). If meta-analysis was performed, describe the model(s), method(s) to identify the presence and extent of statistical heterogeneity, and software package(s) used.	Page 8
	13e	Describe any methods used to explore possible causes of heterogeneity among study results (e.g. subgroup analysis, meta-regression).	Page 8
	13f	Describe any sensitivity analyses conducted to assess robustness of the synthesized results.	Page 8
Reporting bias assessment	14	Describe any methods used to assess risk of bias due to missing results in a synthesis (arising from reporting biases).	Page 8

Certainty assessment	15	Describe any methods used to assess certainty (or confidence) in the body of evidence for an outcome.	Not applicable

Section and Topic	Item #	Checklist item	Location where item is reported
**RESULTS**			
Study selection	16a	Describe the results of the search and selection process, from the number of records identified in the search to the number of studies included in the review, ideally using a flow diagram.	Sup. Fig. 1
	16b	Cite studies that might appear to meet the inclusion criteria, but which were excluded, and explain why they were excluded.	Sup. Table 4
Study characteristics	17	Cite each included study and present its characteristics.	Tables 1-2
Risk of bias in studies	18	Present assessments of risk of bias for each included study.	Sup. Table 5
Results of individual studies	19	For all outcomes, present, for each study: (a) summary statistics for each group (where appropriate) and (b) an effect estimate and its precision (e.g. confidence/credible interval), ideally using structured tables or plots.	Fig. 1-2
Results of syntheses	20a	For each synthesis, briefly summarise the characteristics and risk of bias among contributing studies.	Pages 9-11
	20b	Present results of all statistical syntheses conducted. If meta-analysis was done, present for each the summary estimate and its precision (e.g. confidence/credible interval) and measures of statistical heterogeneity. If comparing groups, describe the direction of the effect.	Pages 9-11
	20c	Present results of all investigations of possible causes of heterogeneity among study results.	Pages 10-11
	20d	Present results of all sensitivity analyses conducted to assess the robustness of the synthesized results.	Sup. Fig. 17-22
Reporting biases	21	Present assessments of risk of bias due to missing results (arising from reporting biases) for each synthesis assessed.	Page 10 and Sup. Fig 13-16
Certainty of evidence	22	Present assessments of certainty (or confidence) in the body of evidence for each outcome assessed.	Not applicable
**DISCUSSION**			
Discussion	23a	Provide a general interpretation of the results in the context of other evidence.	Page 11-14
	23b	Discuss any limitations of the evidence included in the review.	Page 11-14
	23c	Discuss any limitations of the review processes used.	Page 11-14
	23d	Discuss implications of the results for practice, policy, and future research.	Page 11-15
**OTHER INFORMATION**			
Registration and protocol	24a	Provide registration information for the review, including register name and registration number, or state that the review was not registered.	Page 6
	24b	Indicate where the review protocol can be accessed, or state that a protocol was not prepared.	Page 6
	24c	Describe and explain any amendments to information provided at registration or in the protocol.	Page 6
Support	25	Describe sources of financial or non-financial support for the review, and the role of the funders or sponsors in the review.	Page 2
Competing interests	26	Declare any competing interests of review authors.	Page 2
Availability of data, code and other materials	27	Report which of the following are publicly available and where they can be found: template data collection forms; data extracted from included studies; data used for all analyses; analytic code; any other materials used in the review.	Page 16

From: Page MJ, McKenzie JE, Bossuyt PM, Boutron I, Hoffmann TC, Mulrow CD, et al. The PRISMA 2020 statement: an updated guideline for reporting systematic reviews. BMJ 2021;372:n71. doi: 10.1136/bmj.n71. For more information, visit: http://www.prisma-statement.org/.
